# Interferon-stimulated gene PVRL4 broadly suppresses viral entry by inhibiting viral-cellular membrane fusion

**DOI:** 10.1186/s13578-024-01202-y

**Published:** 2024-02-17

**Authors:** Qiaomei Cai, Nina Sun, Yurui Zhang, Jingfeng Wang, Chaohu Pan, Yu Chen, Lili Li, Xiaorong Li, Wancheng Liu, Saba R. Aliyari, Heng Yang, Genhong Cheng

**Affiliations:** 1 National Key Laboratory of Immunity and Inflammation, Suzhou Institute of Systems Medicine, Chinese Academy of Medical Sciences & Peking Union Medical College, Suzhou, 215123 Jiangsu China; 2 Department of Microbiology and State Key Laboratory for Diagnosis and Treatment of Infectious Diseases of the First Affiliated Hospital, Zhejiang University School of Medicine, Hangzhou, Zhejiang China; 3 Clinical Microbiology and Immunology, Affiliated Hospital of Guizhou Medical University, Guiyang, 550004 Guizhou China; 4 Department of Hematology, Qilu Hospital of Shandong University, Jinan, 250000 Shandong China; 5 Department of Microbiology, Immunology and Molecular Genetics, University of California, Los Angeles, CA 90095 USA

**Keywords:** IFN-I, PVRL4, Viral entry, Membrane fusion

## Abstract

**Background:**

Viral infection elicits the type I interferon (IFN-I) response in host cells and subsequently inhibits viral infection through inducing hundreds of IFN-stimulated genes (ISGs) that counteract many steps in the virus life cycle. However, most of ISGs have unclear functions and mechanisms in viral infection. Thus, more work is required to elucidate the role and mechanisms of individual ISGs against different types of viruses.

**Results:**

Herein, we demonstrate that poliovirus receptor-like protein4 (*PVRL4*) is an ISG strongly induced by IFN-I stimulation and various viral infections. Overexpression of PVRL4 protein broadly restricts growth of enveloped RNA and DNA viruses, including vesicular stomatitis virus (VSV), herpes simplex virus 1 (HSV-1), influenza A virus (IAV) and severe acute respiratory syndrome coronavirus 2 (SARS-CoV-2) whereas deletion of PVRL4 in host cells increases viral infections. Mechanistically, it suppresses viral entry by blocking viral-cellular membrane fusion through inhibiting endosomal acidification. The vivo studies demonstrate that *Pvrl4*-deficient mice were more susceptible to the infection of VSV and IAV.

**Conclusion:**

Overall, our studies not only identify *PVRL4* as an intrinsic broad-spectrum antiviral ISG, but also provide a candidate host-directed target for antiviral therapy against various viruses including SARS-CoV-2 and its variants in the future.

**Supplementary Information:**

The online version contains supplementary material available at 10.1186/s13578-024-01202-y.

## Background

Viruses are obligate intracellular pathogens causing a serious threat to human health despite being relatively simple in structure and composition. For example, millions of people suffer from influenza virus infection worldwide, which manifests mild to severe life threating diseases [1]; the transmission of SARS-CoV-2 in human population has resulted in a global pandemic of coronavirus disease 2019 (COVID-19) and caused more than 6 million deaths [2, 3]. A virus has to undergo multiple lifecycle stages including binding, entry, transcription, translation, replication, packaging and egress to produce new virions in host cells [4]. Among them, enveloped viruses characterized by a host-derived lipid membrane are the most significant pathogens throughout human history [5], and they require membrane fusion to enter the host cells. After binding with a target cell surface, enveloped viruses initiate a fusion process with host cell membranes at the cell surface or following endocytosis which ends with transfer of the viral proteins and nucleic acids into the host cell cytoplasm [6]. Entry into cells is an essential step for viruses to generate an effective infection, therefore finding factors limiting viral entry into host cells is crucial to prevent viral infection.

Vertebrates have evolved biological systems to combat invading pathogens and the innate immune system is the first barrier of host defense to against the microbial pathogens invasion. IFNs are the foundation of the host defense to viral infections through binding to the IFNs receptors on the cell surface, which initiate the downstream Janus kinase (JAK)-signal transducer and activator of transcription (STAT) signaling pathway and lead to the induction of a wide array of ISGs [4, 7–11]. The mechanisms behind the antiviral effects of many ISGs have been described. For example, SERTA domain containing 3 (SERTAD3) has an antiviral ability to IAV and Zika virus (ZIKV) by blocking the assembly of viral RNA polymerase complex and inducing proteasomal degradation of ZIKV capsid protein, respectively [12, 13]; Cholesterol-25-hydroxylase (CH25H) broadly inhibits viral entry by production of 25-hydroxycholesterol (25HC) [14–16]; Interferon-induced transmembrane proteins (IFITMs) inhibit infection of diverse enveloped viruses by blocking the fusion of the viral-cellular membrane to hinder the virus entering the cytoplasm [17–20]. So far, about 300 ISGs has been identified based on gene expression studies [21], but only a small number of them have been characterized. Thus, more work is required to elucidate the role and mechanisms of individual ISGs against different types of viruses.

PVRL4, also known as Nectin4, is a type I transmembrane glycoprotein. It is a member of the nectin family that belongs to the family of immunoglobulin-like cell adhesion receptors superfamily and plays a role in cell proliferation, adhesion and migration [22, 23]. PVRL4 protein contains an extracellular region with three immunoglobulin (Ig)-like loops (V, C, C types) that participates in a complex network of homotypic and heterotypic interactions with nectins, a single transmembrane segment, and a cytoplasmic tail that contains a PDZ binding motif and interacts with the adaptor protein afadin [22, 23]. Studies have shown that PVRL4 is abnormally overexpressed in a variety of human cancers, including urothelial cancer, bladder cancer, gastric cancer, thyroid cancer, breast cancer, esophageal cancer, and ovarian cancer [24–27]. Thus, PVRL4 has been defined as a tumor-associated antigen in various cancers and it is a potential cancer therapeutic target [24, 28]. In addition, PVRL4 has been reported to function as a receptor for measles virus by binding to its hemagglutinin (MV-H) [29–31]. However, it is less known for its antiviral activities and mechanisms.

In this study, we characterized PVRL4 as an antiviral ISG that can effectively restrict the infection of multiple enveloped viruses including VSV, HSV-1, IAV and SARS-CoV-2 in vitro. And *Pvrl4*^*−/−*^ mice developed more severe symptoms upon VSV and IAV infection. Furthermore, we found that PVRL4 broadly suppressed viral entry by blocking viral-cellular membrane fusion through inhibiting endosomal acidification.

## Results

### PVRL4 is induced by IFN treatment and viral infection

**Fig. 1:**
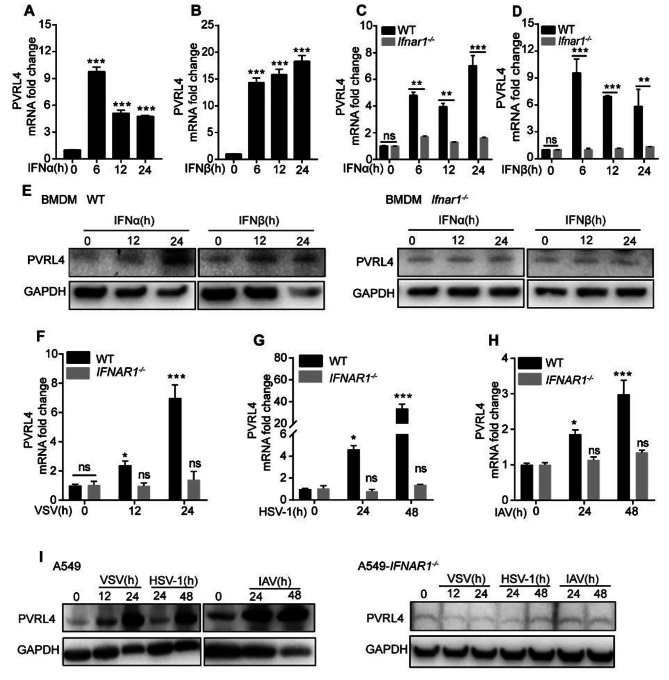
PVRL4 is induced by IFN treatment and viral infection. (**A** and **B**) PVRL4 expression was measured by qRT-PCR in WT BMDMs after treatment with recombinant mouse IFN-α (1000U/mL) (**A**), or IFN-β (1000U/mL) (**B**) for the indicated times. (**C**-**E**) qRT-PCR and western blotting analysis of PVRL4 expression in WT and *Ifnar1*^*−/−*^ BMDMs treated with mouse IFN-α (500U/mL) or IFN-β (500U/mL). (**F**-**I**) qRT-PCR and western blotting analysis of PVRL4 expression in WT and IFNAR1^−/−^ A549 cells infected with VSV, HSV-1 and IAV for the indicated times. Mean ± SEM of three independent experiments. *p < 0.05, **p < 0.01, ***p < 0.001

IFN-I has been recognized as the major antiviral cytokine in vertebrates in response to viral infection [8, 32]. In our previous research, we systematically identified antiviral ISGs containing PVRL4 [21]. Here, to characterize the induction of PVRL4 by IFN-I, we conducted the luciferase reporter plasmids driven by the *PVRL4* promoter which is located 2000 bases upstream of the 5’UTR and found that the promoter of *PVRL4* responded directly to IFN-I treatment (Fig. S1A). Then we stimulated WT and IFNα/β receptor subunit 1 (*Ifnar1*)-deficient murine bone-marrow-derived macrophages (BMDMs) with mouse IFN-α and IFN-β and quantified the mRNA and protein levels by quantitative real-time PCR (qRT-PCR) and western blotting assay, respectively. We found that both PVRL4 mRNA and protein expressions were induced by IFN-α and IFN-β in WT BMDMs but not in the *Ifnar1*^*−/−*^ BMDMs (Fig. 1A-E and Fig. S1B). To identify the expression of PVRL4 in response to viral infection, we evaluated the mRNA levels of PVRL4 in cells infected with VSV, HSV-1 and IAV. The results showed that viral infection increased the amount of mRNA of PVRL4 in A549 cells and HEK293T cells but not in the *IFNAR1*^*−/−*^A549 cells (Fig. 1F-H and Fig. S1C-H). Similarly, the protein expression was also induced by viral infection in A549 cells but not in *IFNAR*^*−/−*^ A549 cells (Fig. 1I and Fig. S1I). These results indicate that PVRL4 is an ISG induced by IFN-I and viral infection in an IFNAR1-dependent manner.

### Overexpression of PVRL4 reduces infections of different types of enveloped viruses in vitro

**Fig. 2:**
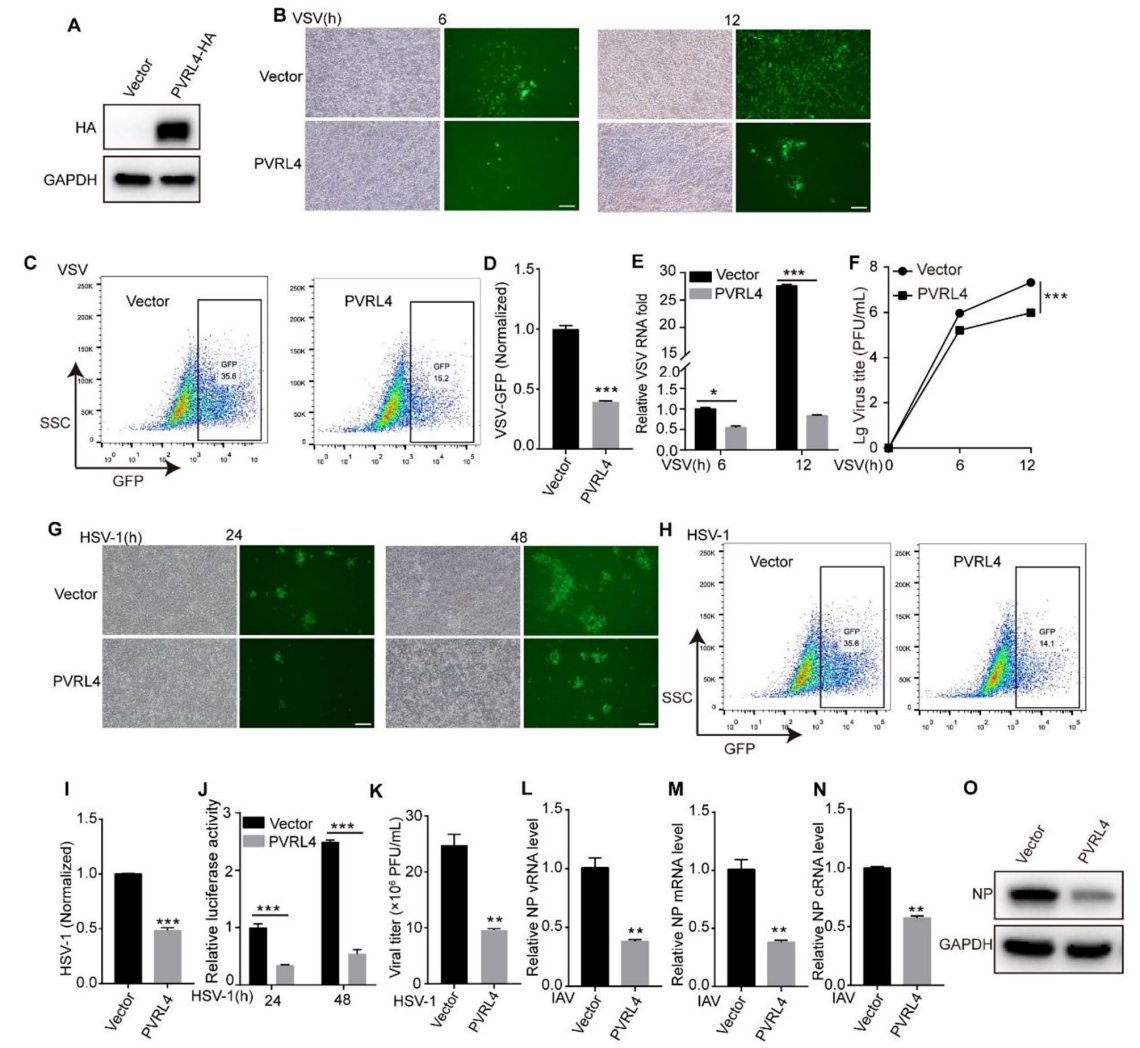
Overexpression of PVRL4 reduces infections of different types of enveloped viruses in vitro. (**A**) HEK293T cells were transfected with PVRL4-HA or HA-expressing plasmids. At 24 h post-transfection, the expression of PVRL4-HA was analyzed by western blotting assay. (**B**-**F**) PVRL4 or control vector-transfected HEK293T cells were infected with VSV (MOI = 0.01). The GFP were visualized by fluorescence microscopy (**B**). The GFP-positive cells were analyzed by flow cytometry (**C**), and the percentages of GFP-positive cells were normalized to the control sample (**D**). The viral M RNA level and vitral titers in the supernatant was measured by qRT-PCR (**E**) and plaque assay (**F**), respectively. Scale bar, 100 μm. The “Lg” means “Log_10_ change”. (**G**-**K**) HEK293T cells were transfected with PVRL4 or vector plasmid for 24 h. Then the cells were infected with HSV-1 (MOI = 0.01) and the GFP was visualized by fluorescence microscopy (**G**). The GFP-positive cells were analyzed by flow cytometry (**H**), and values calculated and normalized in (**I**). The luciferase activity of cells was detected for the indicated times and normalized to the control (**J**). And after 48 h infection, the vitral titers in supernatants were determined by plaque assay (**K**). Scale bar, 100 μm. (**L**-**O**) HEK293T cells were transfected with PVRL4 or vector plasmid, then these cells were infected with IAV (MOI = 0.01) at 24 h post transfection. After 48 h infection, the viral NP vRNA, mRNA and cRNA level were measured by qRT-PCR (**L**-**N**), and viral NP protein expression were measured by western blotting assay (**O**). Mean ± SEM of three independent experiments. *p < 0.05, **p < 0.01, ***p < 0.001

We next determined whether PVRL4 had an antiviral activity against different types of viruses in vitro. HEK293T cells were transfected with PVRL4-HA or HA alone as negative control and the expression of PVRL4 was detected by western blotting assay after 24 h post transfection (Fig. 2A). After that, cells were infected with VSV (Fig. 2B-F), HSV-1 (Fig. 2G-K) and IAV (Fig. 2L-O) at the different multiplicity of infection (MOI) after 24 h post transfection. The results of fluorescence microscopy and flow cytometry clearly showed that overexpression of PVRL4 substantially inhibited VSV-GFP replication relative to cells expressing the control vector (Fig. 2B-D). Similarly, the VSV-M RNA level by qRT-PCR (Fig. 2E) and the viral titers in supernatants by plaque assay (Fig. 2F) also demonstrated that PVRL4 inhibited VSV replication. Similarly, we found that overexpression of PVRL4 could inhibit the replication of HSV-1 by fluorescence microscope and flow cytometry (Fig. 2G-I), detecting the luciferase activity (Fig. 2J) and the plaque assay (Fig. 2K). In addition, we found that the ectopic expression of PVRL4 could suppress the IAV replication by measuring the viral NP vRNA, mRNA and cRNA levels by qRT-PCR (Fig. 2L-N), and the viral NP protein expression by western blotting assay (Fig. 2O). And we found that PVRL4 suppressed the VSV, HSV-1 and IAV infections in a dose-dependent manner (Fig. S2A-G). Interestingly, overexpression of PVRL4 in HeLa cells and HEK293T cells did not seem to inhibit CVB3 infection, a single stranded RNA virus without envelope belonging to the enterovirus (Fig. S3A-E) [33]. Together, these results indicate that overexpression of PVRL4 significantly inhibits infection of multiple enveloped viruses including VSV, HSV-1 and IAV.

### Elevated viral infection in PVRL4-dificient cells

**Fig. 3:**
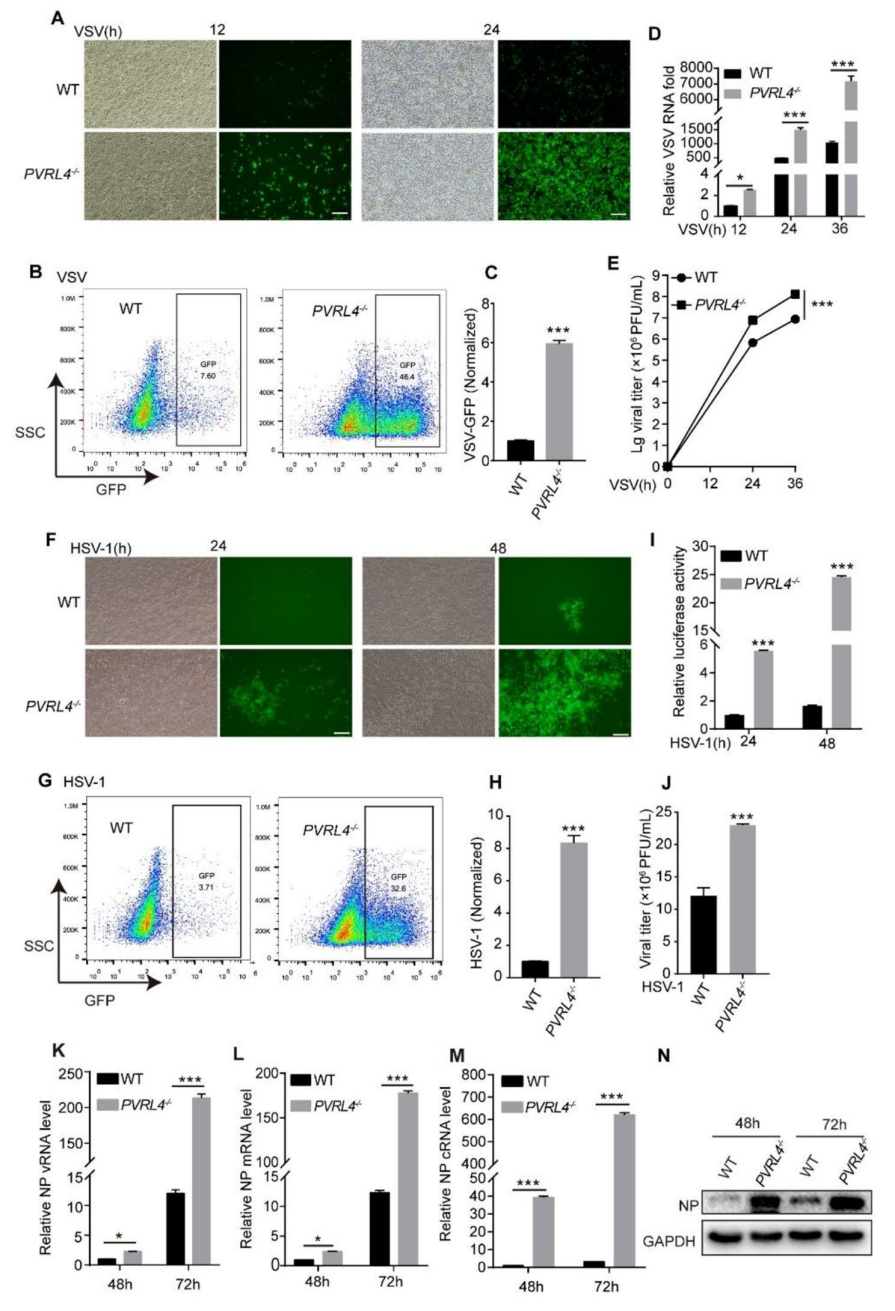
Elevated viral infection in PVRL4-deficient cells. (**A**-**E**) WT and *PVRL4*^*−/−*^HEK293T cells were infected with VSV (MOI = 0.001), and the GFP in the cells were visualized by fluorescence microscopy (**A**). The GFP-positive cells were analyzed by flow cytometry (**B**), and values calculated and presented in (**C**). The viral M RNA level (**D**) and the vitral titers (**E**) were measured by qRT-PCR and plaque assay, respectively, for the indicated times. Scale bar, 100 μm. The “Lg” means “Log_10_ change”. (**F**-**J**) WT and *PVRL4*^*−/−*^HEK293T cells were infected with HSV-1 (MOI = 0.001). The GFP in the cells were measured by fluorescence microscopy (**F**). The GFP-positive cells were analyzed by flow cytometry (**G**), and values calculated and presented in (**H**). And the luciferase activity was measured and normalized to the WT (**I**). And after 48 h infection, the vitral titers in supernatants were determined by plaque assay (**J**). Scale bar, 100 μm. (**K**-**N**) WT and *PVRL4*^*−/−*^HEK293T cells were infected with IAV (MOI = 0.001), and the viral NP vRNA, mRNA and cRNA level (**K**-**M**) and viral NP protein expression (**N**) were measured by qRT-PCR and western blotting assay, respectively, for the indicated times. Data are representative of at least three independent experiments. *p < 0.05, **p < 0.01, ***p < 0.001

To evaluate the effect of endogenous PVRL4 on viral infection in vitro, we generated PVRL4 stable knockout HEK293T cell lines by CRISPR/Cas9 system, which were identified by PCR and western blotting assay (Fig. S4A-C). The PVRL4-deficiency did not influence the cell growth rate (Fig. S4D). Wild-type (WT) and *PVRL4*^*−/−*^ HEK293T cells were infected with VSV (Fig. 3A-E), HSV-1 (Fig. 3F-J) and IAV (Fig. 3K-N). The results of fluorescence microscopy and flow cytometry showed that the number of GFP-positive cells increased in the PVRL4-deficiency cells (Fig. 3A-C). Consistently, qRT-PCR of VSV-M (Fig. 3D) and the viral titers in culture supernatants detected by plaque assay (Fig. 3E) also demonstrated that PVRL4 deficiency significantly enhanced VSV replication. We found that PVRL4-deficient cells had increased the replication of HSV-1 as compared to the parental cells by fluorescence microscope and flow cytometry (Fig. 3F-H), detecting the luciferase activity (Fig. 3I) and the plaque assay (Fig. 3J). In addition, qRT-PCR assays of the IAV NP vRNA, mRNA and cRNA (Fig. 3K-M) and western blotting assay of the IAV NP protein expression (Fig. 3N) showed that the IAV replication rate was increased in *PVRL4*^*−/−*^ HEK293T cells. Consistent with the overexpression of PVRL4, we found that PVRL4 deletion did not increase CVB3 infection (Fig. S3F-G). Thus, our studies demonstrate more robust viral infection in PVRL4-deficient cells, which is consistent with the reduced viral infection in cells overexpressing PVRL4.

### PVRL4 inhibits the entry of different viruses

**Fig. 4:**
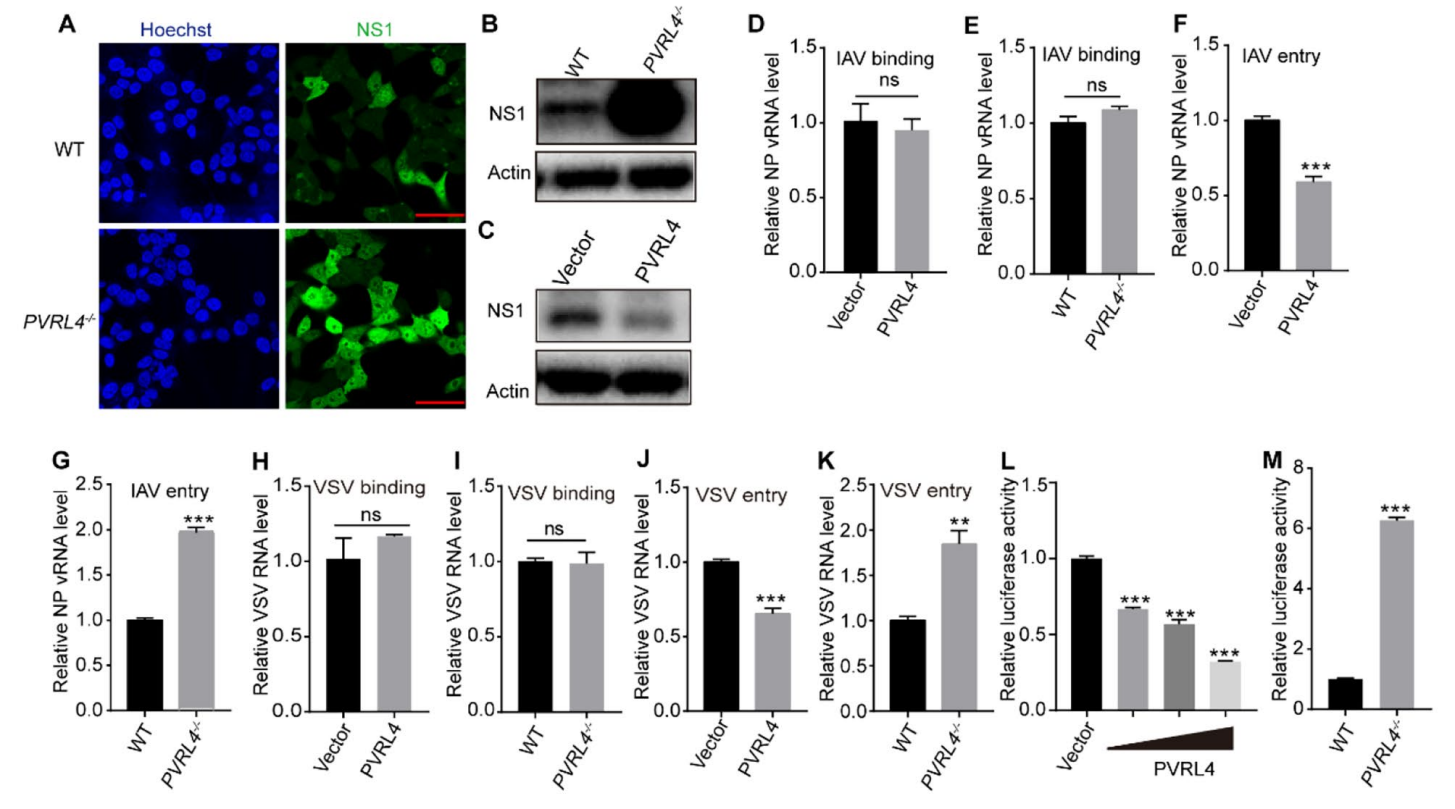
PVRL4 inhibits the entry of VSV and IAV. (**A**-**C**) WT or *PVRL4*^*−/−*^HEK293T cells and HEK293T cells overexpressing PVRL4 or vector were infected with IAV (MOI = 5) for 3 h and processed for immunofluorescence staining (**A**) and western blotting assay (**B** and **C**) with anti-NS1 antibody. (**D** and **E**) PVRL4 or control vector-transfected HEK293T cells and WT or PVRL4^−/−^ HEK293T cells were infected with IAV (MOI = 5) for 1 h at 4℃ and then the cells were harvested to assess the viral NP vRNA copy number through qRT-PCR assay. (**F** and **G**) PVRL4 or control vector-transfected HEK293T cells and WT or *PVRL4*^*−/−*^ HEK293T cells were infected with IAV (MOI = 5) for 1 h at 4℃ and then incubated at 37℃ for 10 min. After that, the cells were washed with acidic-PBS and the internalized viral particles were analyzed by qRT-PCR. (**H**-**K**) For the VSV binding experiment, PVRL4 or control vector-transfected HEK293T cells, and WT or *PVRL4*^*−/−*^ HEK293T cells were infected with VSV (MOI = 5) at 4℃ for 1 h. Cell-surface binding was assessed by determning the viral copy number in the cell lysates through qRT-PCR assay (**H** and **I**). For the VSV entry experiment, after infected at 4℃ for 1 h, the cells were incubated at 37℃ for another 30 min to internalize bound virion before an low pH-PBS wash. The internalized virions were determined by qRT-PCR assay (**J** and **K**). (**L** and **M**) HEK293T cells transfected with PVRL4 or control vector and WT or *PVRL4*^*−/−*^ HEK293T cells were infected with VSV pseudovirus for 24 h. The cell lysates were collected and measured for luciferase activity. Then the results was normalized to the control cells. Mean ± SEM of three independent experiments. *p < 0.05, **p < 0.01, ***p < 0.001, two-tailed Student’s t test

We then explored the mechanism by which PVRL4 inhibits viral infection. To address whether PVRL4 played a role in the early stages of viral life cycle, we infected HEK293T cells with IAV for 3 h before immunofluorescence staining and western blotting assay of viral NS1. We found that PVRL4 deficiency increased the IAV infection (Fig. 4A and B), and overexpression PVRL4 inhibited the IAV infection (Fig. 4C). These data suggested that PVRL4 was involved in the early stages of viral life cycle: binding, entry, uncoating, nuclear import of vRNP, transcription, replication and translation. To further dissect the role of PVRL4 in early viral infection, we examined the effect of PVRL4 on the binding and entry of IAV and VSV. We found that PVRL4 affected the entry but not the binding step of IAV infection (Fig. 4D-G). Similarly, we found that PVRL4 effectively suppressed the entry of VSV but not the binding (Fig. 4H-K). In addition, we took advantage of the VSV-G pseudovirus system which has only a single-round infection to further investigate the viral entry process. Quantification of luciferase activity showed that PVRL4 inhibited the VSV pseudovirus infection (Fig. 4L and M). Taken together, PVRL4 inhibits the entry step of VSV and IAV infections.

### PVRL4 inhibits viral protein-mediated cell-cell membrane fusion

**Fig. 5:**
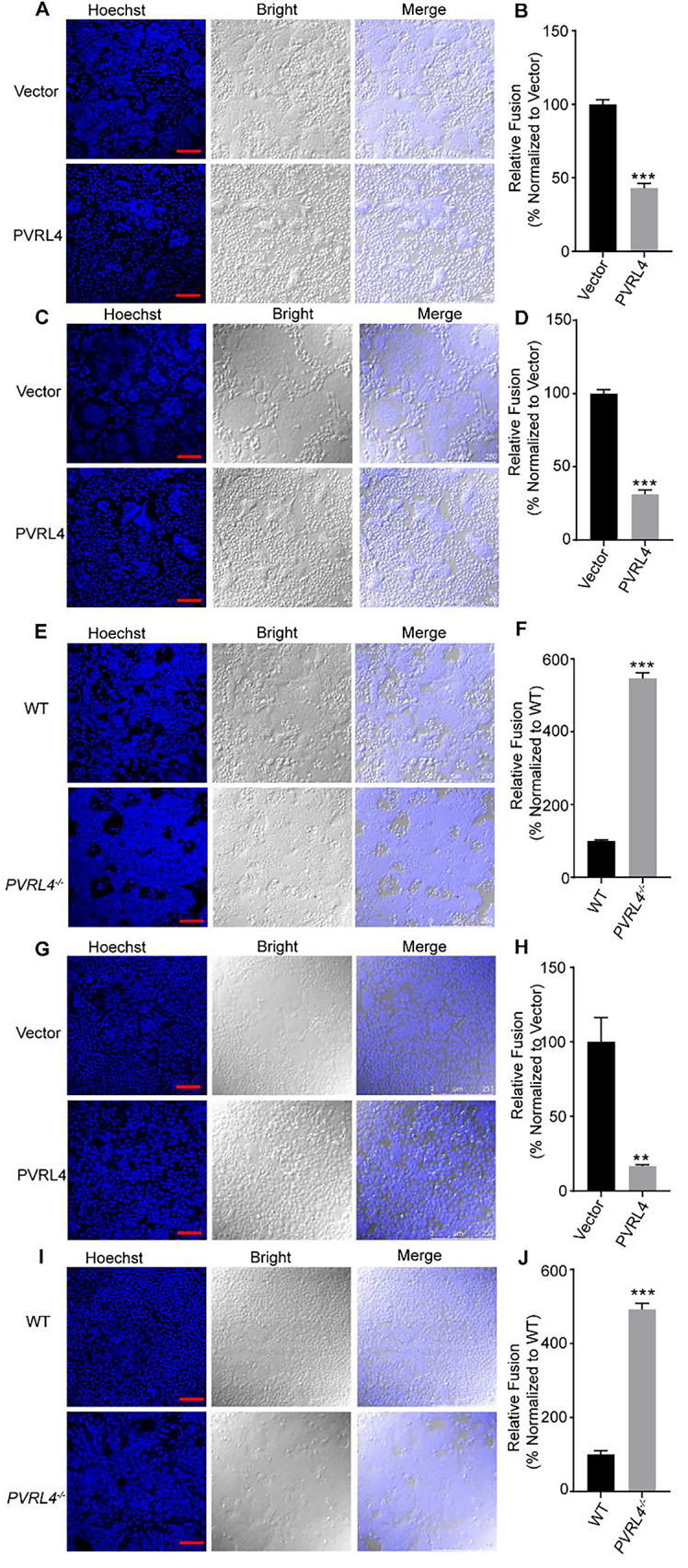
PVRL4 inhibits viral-cellular membrane fusion. (**A** and **B**) HEK293T cells were contransfected with pMD2G, either PVRL4 or vector for 24 h and treated with low-pH PBS. And the syncytia formation was visualized by laser scanning confocal microscope, scale bar, 100 μm. (**C** and **D**) HEK293T cells expressing PVRL4 or vector were co-cultured with HEK293T cells expressing pMD2G at the ratio of 1:1 for 6 h before treated with acidic PBS. Note the formation of cell-cell fusion, scale bar, 100 μm. (**E** and **F**) WT or *PVRL4*^*−/−*^HEK293T cells were transfected with pMD2G. At 24 h post-transfection, the cells were treated with low-pH PBS and the syncytium formation was visualized by laser scanning confocal microscope, scale bar, 100 μm. (**G** and **J**) PVRL4-overexpressing or control vector A549 cells and WT or *PVRL4*^*−/−*^A549 cells were infected with HSV-1 (MOI = 0.01) for 24 h. The HSV-1 viral protein-mediated cell-cell membrane fusion was visualized by laser scanning confocal microscope, scale bar, 100 μm. Relative fusion was determined by normalizing the number of nuclei per syncytia under the experimental conditions to the control. Mean ± SEM of three independent experiments. *p < 0.05, **p < 0.01, ***p < 0.001, two-tailed Student’s t test

To further dissect the role of PVRL4 in viral entry, we examined the effect of PVRL4 on VSV-G protein and HSV-1 virus mediated membrane fusion. VSV infection depends on the fusion of viral and cellular membranes, which is mediated by viral spike glycoprotein G at the low pH environment [34, 35]. The cell-to-cell fusion assay was conducted in cells with co-expression of VSV-G and PVRL4 or vector, independent of viral infection. We found that overexpression the PVRL4 blocked the VSV-G protein mediated cell-cell membrane fusion (Fig. 5A and B). To mimic the viral and cellular membrane fusion, we transfected VSV-G into HEK293T cells as the donor cells and co-cultured with HEK293T cells expressing PVRL4 or vector as the recipient cells. As expected, we observed few VSV-G protein-mediated syncytia formation when co-cultured with PVRL4 overexpressing cells (Fig. 5C and D). Consistent with the above results, VSV-G protein-mediated membrane fusion was increased in PVRL4-deficient cells as compared with WT cells (Fig. 5E and F). Next, we constructed A549-PVRL4 cells that stably overexpressed PVRL4 and used these cells to infect HSV-1 (MOI = 0.01). At 48 h post-infection, we found that the overexpression of PVRL4 significantly decreased the membrane fusion (Fig. 5G and H). Furthermore, we monitored the efficiency of HSV-1 viral protein-mediated membrane fusion in WT and *PVRL4*^*−/−*^ A549 cells. The results also showed that the fusion was increased in *PVRL4*^*−/−*^A549 cells (Fig. 5I and J). PVRL4 is a single-pass type I membrane protein and has been found expressed in the plasma membrane [23]. To define the underlying antiviral mechanisms of the PVRL4 further, we investigated the PVRL4 cellular localization. Confocal microscopy showed that PVRL4 was expressed at the cell surface and a small number of PVRL4 was located on the early endosome once viral infection (Fig. S5A and B). Since VSV, HSV-1 and IAV can entry cells through endocytic pathway, we detected the endosomal acidification which is necessary for viral entry through endocytic pathway [36–39]. We found that PVRL4-decifient increased the level of endosomal acidification (Fig. S5C). And after viral infection, the number of acidified endosomes increased in both cells, but the endosomal acidification in *PVRL4*^*−/−*^ A549 cells remained significantly higher than in WT A549 cells (Fig. S5D). In conclusion, our results reveal that PVRL4 inhibits viral protein-mediated cell-cell membrane fusion and the endosomal acidification.

### *Pvrl4*-decifient mice are more sensitive to viral infection

**Fig. 6:**
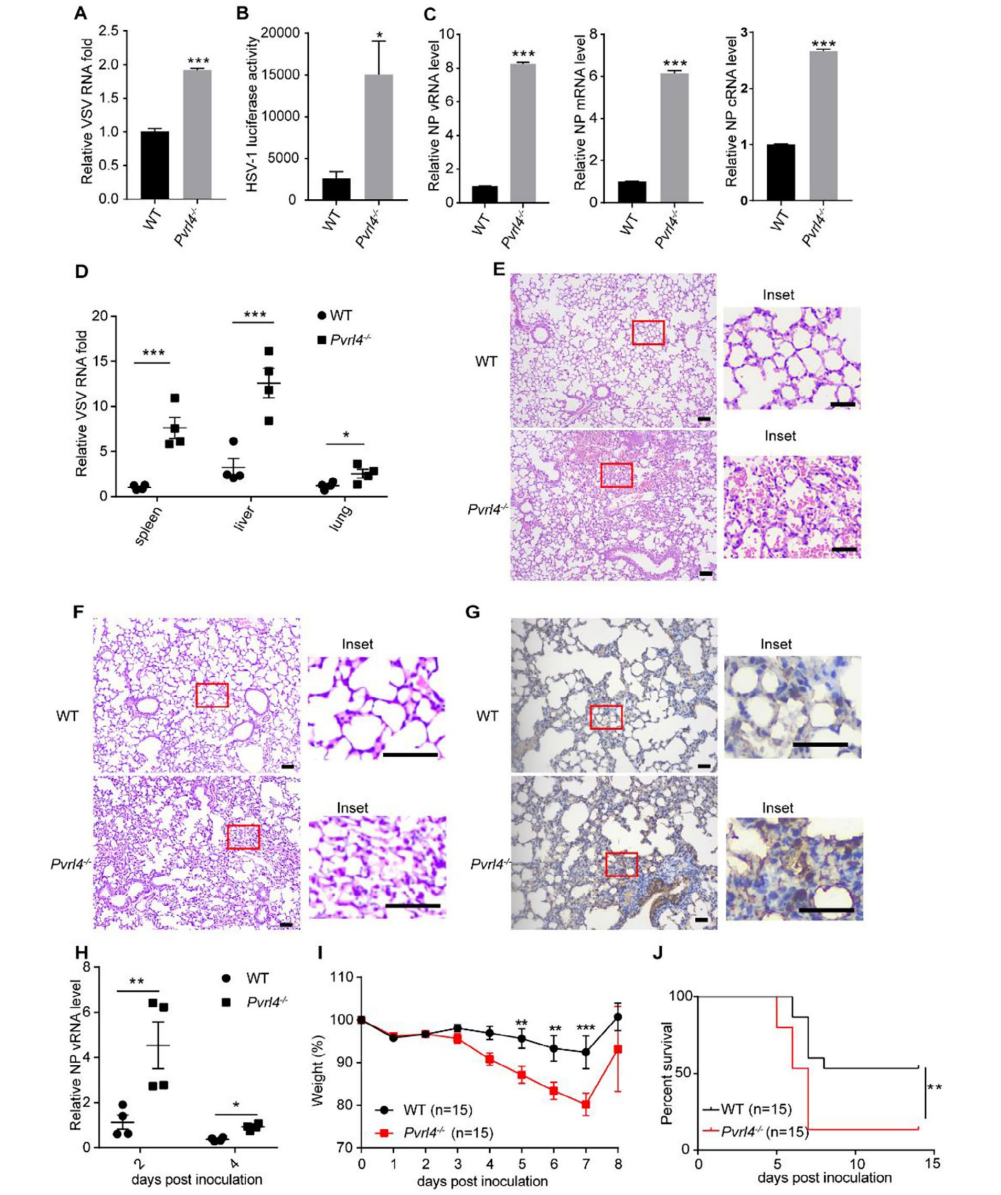
PVRL4-decifient mice are more sensitive to viral infection. (**A**-**C**) BMDMs derived from WT and *Pvrl4*^*−/−*^ mice were infected with VSV (MOI = 1), HSV-1 (MOI = 10) and IAV (MOI = 5). And the viral M RNA level of VSV were measured by qRT-PCR (**A**), the luciferase activity of HSV-1 was measured (**B**) and the IAV NP vRNA, mRNA and cRNA levels were measured by qRT-PCR (**C**). (**D** and **E**) WT and *Pvrl4*^*−/−*^ mice were intraperitoneally injected with VSV (1 × 10^8^PFU/g) (*n* = 4). The VSV RNA expression in organs was detected by qRT-PCR after infection for 36 h (**D**). The lungs were stained with H&E (**E**). Scale bar, 100 μm; inset scale bar, 100 μm. (**F**) H&E staining of lungs of WT and *Pvrl4*^*−/−*^ mice infected with 10,000 PFU of IAV at 2 days post-infection. Scale bar, 100 μm; inset scale bar, 100 μm. (**G**) Immunohistochemical detection of NP in the lungs of WT and *Pvrl4*^*−/−*^ mice infected with IAV at 2 days post-infection. Scale bar, 50 μm; inset scale bar, 50 μm. (**H**-**J**) 6 to 8 weeks mice of WT and *Pvrl4*^*−/−*^ were infected with IAV (10000PFU) intranasally. The RNA level of NP in the lung were measured by qRT-PCR at 2 and 4 days post-infection (H, *n* = 4). And the bodyweight loss (**I**) and survival (**J**) were monitored for 8 days and 14 days, respectively (*n* = 15)

As our in vitro data indicated that PVRL4 restricted viral infection, we next investigated the role of PVRL4 during viral infections in vivo. We generated *Pvrl4*^*−/−*^ mice using CRISPR/Cas9 technology by Biocytogen (Beijing, China). *Pvrl4*^*−/−*^ mice were normal in weight and did not display any physical or behavioral abnormalities compared to WT mice (Fig. S6). We generated BMDMs from WT or *Pvrl4*^*−/−*^ mice. After challenging with VSV, HSV-1 and IAV, we found that knockout of *Pvrl4* significantly increased VSV, HSV-1 and IAV viral burdens (Fig. 6A-C). After intraperitoneal injection with VSV (1 × 10^8^PFU/g), we found that the VSV RNA levels in organs (spleen, liver, and lung) of *Pvrl4*^*−/−*^ mice was significantly higher than those in WT mice (Fig. 6D). Consistently, *Pvrl4*^*−/−*^ mice also displayed more severe pathologic changes in the lungs compared with WT mice (Fig. 6E). In addition, WT and *Pvrl4*^*−/−*^mice were intranasally infected with WSN virus (10,000 PFU). Histopathological analysis indicated that *Pvrl4*^*−/−*^ mice showed more severe lung tissue damage and inflammation (Fig. 6F). Further, immunohistochemical staining of viral protein NP revealed elevated levels of viral antigens in *Pvrl4*^*−/−*^ mice (Fig. 6G). The NP vRNA level in the lung tissues of *Pvrl4*^*−/−*^ mice were also significantly higher than those of WT mice (Fig. 6H). More importantly, compared with WT mice, the infected *Pvrl4*^*−/−*^ mice showed more significant weight loss and an increased mortality rate (Fig. 6I and J). These results indicate that *Pvrl4* plays an important role in controlling viral infection in vivo.

### PVRL4 is an anti-SARS-CoV-2 ISG that can inhibit its spike-mediated membrane fusion and viral entry

**Fig. 7:**
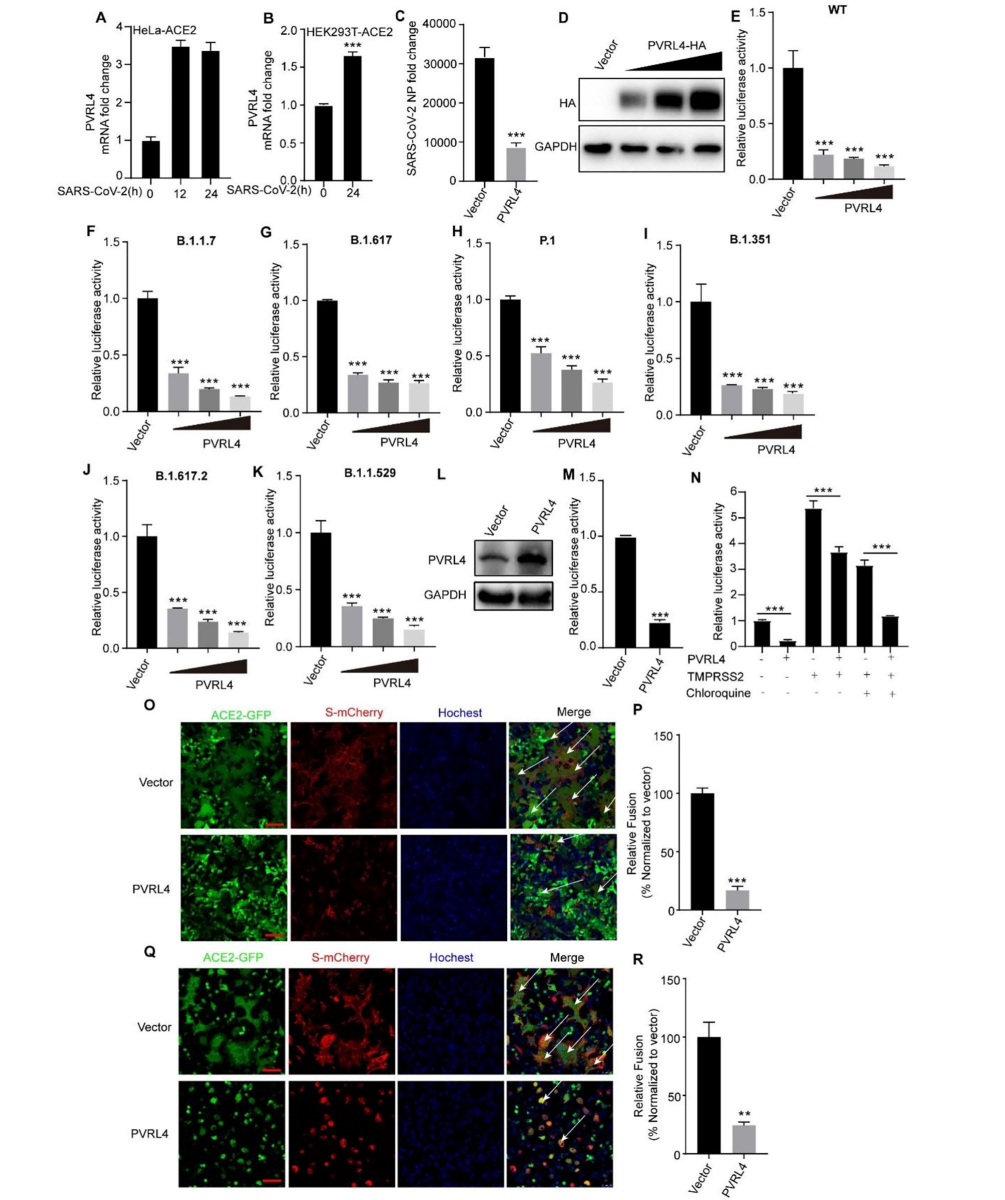
PVRL4 is an anti-SARS-CoV-2 ISG that can inhibit its spike-mediated membrane fusion and viral entry. (**A**) qRT-PCR analysis of PVRL4 expression in Hela-ACE2 infected with SARS-CoV-2 or uninfected for the indicated times. (**B**) qRT-PCR analysis of PVRL4 expression in HEK293T-ACE2 infected with SARS-CoV-2 pseudovirus for the indicated time. (**C**) qRT-PCR analysis of SARS-CoV-2 NP RNA levels in HEK293T-ACE2 cells transfected with PVRL4 or vector plasmids for 30 h, followed by infection for 24 h with SARS-CoV-2 (MOI = 0.25). (**D**) Western blotting analysis of lysates from HEK293T-ACE2 cells transfected with increasing amounts of PVRL4-HA plasmids (0, 100, 300, 500ng). (**E**-**K**) HEK293T-ACE2 cells were transfected with PVRL4 or control vector plasmids. After 24 h transfection, the cells were infected with SARS-CoV-2 pseudovirus (**E**) or various variants (**F**-**K**). Then luciferase activity was performed to determine the pseudovirus quantity. (**L**) Western blotting analysis of lysates from A549-ACE2 cells that overexpressed PVRL4 or not. (**M**) A549-ACE2 cells that overexpressed PVRL4 or not were infected SARS-CoV-2 pseudovirus. After 24 h infection, the luciferase activity was performed to determine the pseudovirus quantity. (**N**) HEK293T-ACE2 cells were co-transfected with TMPRSS2 expression plasmid and PVRL4 or vector control plasmid for 24 h. Cells were treated with 100µM chloroquine or DMSO before SARS-CoV-2 pseudovirus infection. The pseudovirus infection was quantified by luciferase activity at 24 h post-infection. (**O** and **P**) HEK293T-ACE2 cells were cotransfected with SARS-CoV-2-spike-mCherry and PVRL4 or vector for 24 h. The white arrows highlight the syncytia formation. Scale bars, 100 μm. Relative membrane fusion was determined by normalizing the number of nuclei per syncytia to the vector cells set to 100% (**P**). (**Q** and **R**) PVRL4 or control vector-transfected HEK293T-ACE2 cells were co-cultured with HEK293T cells expressing spike-mCherry at the ratio of 1:1, and then the syncytium formation was visualized by laser scanning confocal microscope at the 6-8 h post co-culture (**Q**). Scale bars,100 μm. White arrows indicated syncytia. Relative membrane fusion was determined by normalizing the number of nuclei per syncytia to the vector cells set to 100% (**R**). Mean ± SEM of three independent experiments. *p < 0.05, **p < 0.01, ***p < 0.001, two-tailed Student’s t test

The previous results indicated that PVRL4 broadly suppressed viral infection by inhibiting viral-cellular membrane fusion, so we evaluated whether PVRL4 can inhibit the infection of SARS-CoV-2, a newly prevalent enveloped RNA virus. To characterize the induction of PVRL4 by SARS-CoV-2, we infected Hela-ACE2 cells that stably expressed the angiotensin-converting enzyme 2 (ACE2) with SARS-CoV-2. We found that SARS-CoV-2 infection increased the mRNA levels of PVRL4 in Hela-ACE2 cells (Fig. 7A). Similarly, we also found that the mRNA levels of PVRL4 was increased in HEK293T-ACE2 cells when infected with SARS-CoV-2 pseudovirus (Fig. 7B). Then we sought to determine whether PVRL4 suppressed the infection of SARS-CoV-2. HEK293T-ACE2 cells overexpressed PVRL4 or transfected with vector were infected with SARS-CoV-2 for 24 h. The SARS-CoV-2 NP RNA levels in cells were determined by qRT-PCR and the result demonstrated that PVRL4 inhibited SARS-CoV-2 infection (Fig. 7C). Then we transfected HEK293T-ACE2 cells with increasing amounts of PVRL4-HA plasmids and detected the protein by western blotting assay (Fig. 7D). HEK293T-ACE2 cells overexpressing PVRL4 or vector were infected with WT and variant SARS-CoV-2 pseudovirus including B.1.1.7 lineage first reported in the United Kingdom, the B.1.617 lineage in India, the P.1 lineage in Brazil, the B.1.351 lineage in South Africa and the Omicron (B.1.1.529) which not only spread rapidly but also exhibit increased resistance to immunity induced by the vaccination of WT [40, 41]. We found that their infections were suppressed in PVRL4-overexpressing HEK293T-ACE2 cells (Fig. 7E-K). Beyond that, we also constructed A549-ACE2 cell lines that stably overexpressing PVRL4 which were a type of lung epithelial cells and were more relevant to SARS-CoV-2 (Fig. 7L). And we found that overexpression of PVRL4 suppressed SARS-CoV-2 pseudovirus infection in A549-ACE2 cells (Fig. 7M). Consistently, infections of these pseudoviruses were enhanced in *PVRL4*^*−/−*^ HEK293T cells compared to the WT cells (Fig. S7A-G). These results indicated that PVRL4 could affect the entry of SARS-CoV-2 through endocytic pathway. Besides that, when transmembrane protease, serine2 (TMPRSS2) is present at the cell surface, SARS-CoV-2 can enter host cells through plasma membrane fusion pathway [42, 43]. Then we investigated if PVRL4 suppressed SARS-CoV-2 entry through plasma membrane fusion pathway. HEK293T-ACE2 cells were co-transfected with TMPRSS2 expression plasmid and PVRL4 or vector control plasmid treated with chloroquine or DMSO before SARS-CoV-2 pseudovirus infection. As demonstrated in Fig. 7N, when TMPRSS2 was expressed, overexpression of PVRL4 also inhibited the viral entry mediated by SARS-CoV-2-S protein. And PVRL4 also inhibited the SARS-CoV-2 entry on the TMPRSS2-overexpressed HEK293T-ACE2 cells when the cells were treated with chloroquine which can block endosomal acidification that is necessary for endocytic pathway [44, 45]. These results indicated that PVRL4 also suppressed SARS-CoV-2 entry through plasma membrane fusion pathway. These results indicated that PVRL4 could affect the entry of SARS-CoV-2. We next examined the effect of PVLR4 on SARS-CoV-2 spike-mediated membrane fusion. We co-transfected SARS-CoV-2 spike-mCherry and PVRL4 or vector into HEK293T-ACE2 cells and found that PVRL4 ectopic expression substantially reduced syncytia formation (Fig. 7N and O). We also used a co-culture system of HEK293T-spike-mCherry donor cells and HEK293T-ACE2 acceptor cells with or without overexpressing PVRL4 at the ratio of 1:1. As expected, we observed that PVRL4 overexpression in “recipient” cells reduced the formation of large syncytia (Fig. 7P and Q). Collectively, our results support a model that PVRL4 inhibits SARS-CoV-2 infection through suppressing SARS-CoV-2 spike protein-mediated viral and cell membrane fusion.

## Discussion

The IFNs-mediated innate immune response is inherent in the genomes and provides a strong first line of defense against invading pathogens. Once pathogens invade, the innate immune system utilizes a limited number of germline-encoded pattern-recognition receptors (PRRs) to recognize the molecular structures that are broadly shared by pathogens, called pathogen-associated molecular patterns (PAMPs) [46, 47]. Upon recognition of PAMPs, PRRs initiate a cascade of signaling programs to promote IFNs production. Following pathogens detection and subsequent IFNs production, the IFNs bind to cell surface receptors and initiate a cascade of reactions through JAK-STAT pathway, leading to the induction of ISGs which exert antiviral activities [9, 48]. So far, hundreds of ISGs with antiviral activity have been found, and we need to better understand the antiviral mechanisms of individual ISG in order to inform the rational design of antiviral drugs [21]. And our present study showed that an IFN I-inducible gene, PVRL4, was robustly and lastingly upregulated in host cells treated by either IFN-I or numerous viral infections in an IFNAR1-dependent manner.

PVRL4 is a member of the nectin family and is considered as a tumor-associated antigen with pro-cancer properties in various cancers. It has been identified as an epithelial receptor for several viruses, including measles [29, 30], canine distemper virus [49] and phocine distemper virus [50]. Therefore, PVRL4 has implications for ongoing measles-virus-based oncolytic clinical trials. In contrast to promoting measles-virus infection, we found that PVRL4 possessed antiviral activity against a wide range of enveloped RNA and DNA viruses, containing VSV, HSV-1, IAV and SARS-CoV-2 in vitro. More importantly, we have demonstrated that *Pvrl4*-deficient mice were more susceptible to the infection of VSV and IAV compared with WT mice. Further studies are required for understand the opposite roles of PVRL4 as a receptor for measles but as an antiviral ISG in host innate response.

Virus entry, including attachment and penetration into the host target cells, is the first step of the virus life cycle and is also a crucial aspect of infection. Upon encountering a target cell, virions adhere to the cell surface and begin fusion with host cell membrane or following endocytosis [51]. The occurrence of membrane fusion depends on the fusion proteins (also known as viral glycoproteins) which are encoded by virus and expose on their surface. So far, few ISGs have been identified that restrict viral-cellular membrane fusion, for example, IFITMs, CH25H, lymphocyte antigen 6 complex, locus E (LY6E) [14, 15, 52, 53]. Here, we have presented evidence that PVRL4 inhibited viral entry of multiple different types of viruses. We have further shown that PVRL4 blocked viral protein-mediated membrane fusion through detecting the VSV-G protein and HSV-1 viral protein-mediated syncytial formation. Confocal microscopy showed that PVRL4 was expressed at the cell surface and a small number of PVRL4 was located on the early endosome once viral infection. And we also found that PVRL4-decifient increased the level of endosomal acidification in the presence and absence of viral infection. However, the molecular mechanisms responsible for PVRL4-mediated the level of endosomal acidification as well as its relationship with other viral entry inhibitors such as IFITMs, CH25H and LY6E remain to be elucidated.

Recently, SARS-CoV-2, a coronavirus, is discovered to cause the respiratory disease known as COVID-19 [54, 55]. It is highly contagious among human populations, and its spread has led to a global pandemic with more than 6.9 million of deaths as of May 2023 (https://covid19.who.int). Due to the serious impact of SARS-CoV-2 on global health, it is critical to search for molecules that host protect against viral infection and apply this knowledge to develop new strategies in the prevention and treatment of COVID-19 related diseases. In this study, we have found that PVRL4 was significantly upregulated in Hela-ACE2 cells and HEK293T-ACE2 cells infected by SARS-CoV-2 and it inhibited SARS-CoV-2 infection. SARS-CoV-2 can enter host cells through endocytic pathway and plasma membrane fusion pathway, and we found that PVRL4 inhibited the entry of SARS-CoV-2 as well as various variants through both pathways. Further mechanistic studies revealed that PVRL4 suppressed SARS-CoV-2 spike-protein-mediated membrane fusion.

## Conclusions

**Fig. 8:**
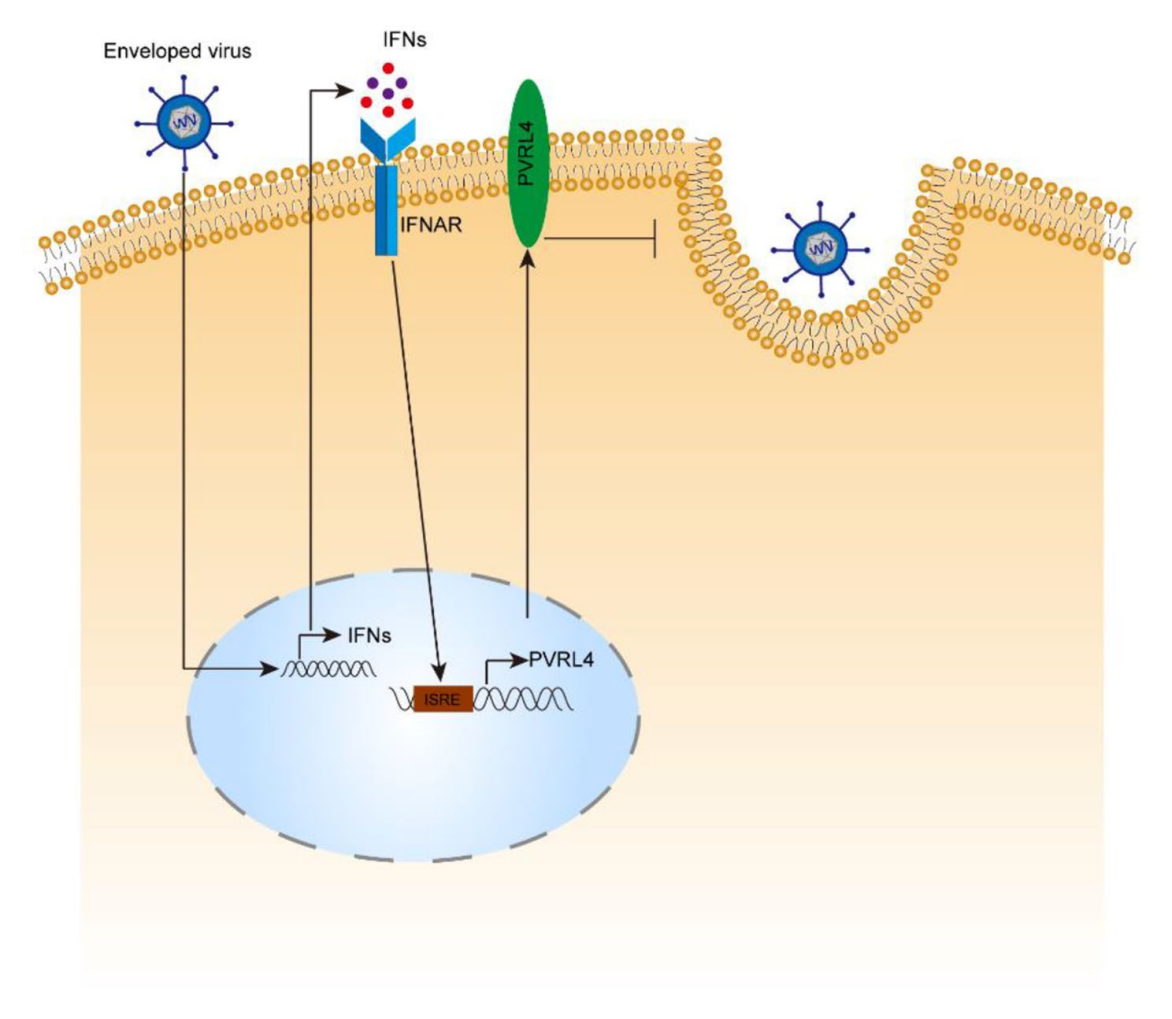
Model of PVRL4-mediated restriction of viral infection. Viral infection elicits the IFN-I response in host cells. And IFN-I binds to the IFNAR1/IFNAR2 heterodimers, subsequently activates a series of signaling pathways to induce the expression of PVRL4. As an ISG, PVRL4 protein broadly restricts growth of enveloped RNA and DNA viruses, including VSV, HSV-1, IAV and SARS-CoV-2, by inhibiting viral entry through blocking viral-cellular membrane fusion

In conclusion, our present study showed that PVRL4 was elevated in host cells treated by either IFN-I or viral infections including VSV, HSV-1, IAV and SARS-CoV-2 in an IFNAR1-dependent manner. As an antiviral ISG, we confirmed that PVRL4 restricted VSV, HSV-1, IAV and SARS-CoV-2 infections in vitro. More importantly, *Pvrl4*^*−/−*^ mice developed enhanced viral proliferation and more severe symptoms upon VSV and IAV infection. Our mechanistic studies showed that PVRL4 suppressed the viral entry by blocking the viral protein-mediated membrane fusion through inhibiting the endosomal acidification (Fig. 8). Viral-cellular membrane fusion is a complex process, and at the current stage, it is not clear how PVRL4 affects viral-cellular membrane fusion and endosomal acidification, how PVRL4 antiviral activity is regulated or whether other co-factors interact with PVRL4. Nonetheless, our studies have demonstrated that PVRL4 is an antiviral ISG in host innate immune defense against multiple different types of viruses and further studies of this molecule may lead to development of potential agents for antiviral therapy against various viruses including SARS-CoV-2 and its variants.

## Materials and methods

### Cells, viruses and reagents

HEK293T, A549, Vero and Hela cells were purchased from American Type Culture Collection (ATCC). The *IFNAR1*^*−/−*^ A549 cells and *PVRL4*^*−/−*^ A549 cell lines were generated by CRISPR-Cas9 technology by our lab in the past [56]. The A549-ACE2 cells were a gift of Professor Zhou Zhuo (Suzhou Institute of System Medicine, China). All the cells were cultured in standard DMEM with 10% fetal bovine serum (FBS), Penicillin (100 Unit/ mL) and Streptomycin (100 µg/mL) and grown at 37℃ in 5% CO_2_. Influenza A/WSN/33 virus, VSV, and HSV-1 were used in our previous research [12, 57, 58]. The HSV-1 with luciferase was kindly provided by Dr. Chunfu Zheng (Fujian Medical University, China). In briefly, the genome of the HSV-1 F strain was cloned as an infectious bacterial artificial chromosome (BAC) clone without any deletions of the viral genes, and a firefly luciferase cassette was inserted to generate a novel luciferase-expressing HSV-1 BAC. The recombinant HSV-1 BAC Luc behaved indistinguishably from the wild-type virus and the luciferase reporter gene can make the quantification of HSV-1 [59]. The luciferase activity was measured with a firefly luciferase substrate kit (Promega, E1960) according to the manufacturer’s protocol. In briefly, the cells infected HSV-1 were washed by PBS, and then were lysed in 1× passive lysis buffer for 30 min. The supernatant was used to detect the luciferase using luciferase substrate kit on the machine of SpectraMax. Mouse macrophage colony-stimulating factor (M-CSF) was purchased from PeproTech. Recombinant mouse IFN-α was purchased from Pbl assay science and recombinant mouse IFN-β was purchased from R&D systems. Wheat Germ Agglutinin (WGA, Red, Invitrogen, W11262) and pHrodo Red dextran (Invitrogen, P10361) were purchased from Invitrogen. Rabbit anti-GAPDH (CST, 5174 S), rabbit anti-PVRL4 (proteintech, 21903-1-AP), mouse anti-Rab5A (CST, 46,449), mouse anti-influenza A nucleoprotein (GeneTex, GTX629633), and rabbit anti-influenza A virus NS1 (nonstructural protein) antibody (GeneTex, GTX638102) were used at the appropriate dilutions.

### Dual-luciferase reporter system

Primers containing XhoI and HindIII sites were used to amplify PVRL4 promoter which is located 2000 bases upstream of the 5’UTR. Then the amplified product was cloned into the luciferase reporter vector pGL4.0 (Addgene, 84,924) which was linearized by XhoI and HindIII. And pGL4.0-PVRL4 promoter and Renilla luciferase plasmid were co-transfected into HEK293T cells using Lipfectamine 3000 (Invitrogen, L3000075). After 24 h transfection, the cells were stimulated by IFN-α (1000U/mL). After 24 h, firefly luciferase and Renilla luciferase activities were detected using a dual luciferase reporter system (Promega, E2940). The ratio of firefly luciferase activity to Renilla luciferase activity was calculated.

### Mice

*Pvrl4*-knockout mice were generated by Biocytogen (Beijing, China) using the CRISPR/Cas9 based EGE system (EGE-YRW-001). Exons 2–7 were deleted according to the structure of the *Pvrl4*. Two single guide RNAs (sgRNAs) were designed to target the introns of both sides of the deleted sequence. Briefly, *in vitro-*synthesized Cas9 mRNA and two small sgRNAs were co-injected into mouse zygotes and the zygotes were then planted into pseudopregnant female mice. Mouse genomic DNA was extracted from the tail tip of 4-week-old mice by KAPA Express Extract DNA Extraction Kit and then was amplified by PCR to identify the genotypes (Forward 5’-TGTTCCCAAGGATGGACCTTACCCT-3’, Reverse5’-TTGTCCTTGAGACTATCAGGGTGGC-3’). Male WT and *Pvrl4*^*−/−*^ littermates of 6-8-week-old were used for all experiments. All mice were raised under specific pathogen-free conditions at the Animal center of Suzhou Institute of Systematic Medicine. All animal experiments were performed according to the protocols approved by the Institutional Animal Care and Use Committee (IACUC) of Suzhou Institute of System Medicine (ISM-IACUC-20,230,007).

### Primary cells of BMDM

Bone marrow was harvested from 6 to 8 weeks WT or *Ifnar1*^*−/−*^ C57BL/6J mice and differentiated in DMEM with 10% fetal bovine serum (Gibco) and 20ng/mL of M-CSF for 7 days. On day 3, the cells were added 500uL supplemental medium of DMEM with 10% FBS containing 20ng/mL of M-CSF. On day 7, the cells were stimulated with recombinant mouse IFN-α or IFN-β. Total cellular RNA was extracted using the RNA-Quick Purification Kit (UUBIO) according to the manufacturer’s protocol.

### VSV and HSV-1 Plaque Assay

VSV and HSV-1 plaque assay was conducted in Vero cells. In brief, the Vero cells were seeded in the 12-well plates for 12 h. Supernatants from infected cells for the indicated times were serially diluted and infected on Vero cells for 1 h. The cells were then covered with growth medium containing 2%FBS,1%PS, 1% low-melting point agarose (VMR life science, 0815-100G). Plaques were stained with crystal violet 0.5% (m/v) in 20% ethanol (v/v) and were counted.

### Gene knockout by the CRISPR/Cas9 system

To knockout Pvrl4 on HEK293T cell lines, two small guide RNAs (sgRNAs) (~ 200 bp gap sequence) targeting the Pvrl4 genes were designed and cloned into sgRNA expression vectors under the control of human U6 promotor. HEK293T cells were co-transfected with sgRNAs and Cas9 expression plasmids, followed by puromycin selection, as described previously [60, 61]. Single clones were isolated by fluorescence-activated cell sorting (FACS) and confirmed by PCR genotyping and sequencing.

### Stable cell line generation

To create a stable cell line for PVRL4 expression, PVRL4 was cloned into the pMXsIG-IgkFLAG vector and co-transfected into HEK293T cells with VSV glycoprotein and pCpG helper plasmids. At the transfection for 48 h, the culture supernatant was collected and filtered with 0.45 μm strainer and then added into A549 cells or A549-ACE2 cells for infection. The cells were collected after infection for 48 h, and the PVRL4-overexpressing cells were then sorted by FACS. HEK293T cells that stably express human ACE2 (HEK293T-ACE2) were established as described [62].

### RNA isolation, reverse transcription and qPCR

Total cellular RNA was extracted using the PureLink RNA Extraction Kit (Thermo Fisher Scientific) following the manufacturer’s instructions. cDNA was synthesized using PrimeScript™ II 1st Strand cDNA Synthesis Kit (TaKaRa). Primers used to amplify corresponding genes are listed on the Table S1. The qPCR was performed using SYBR Green qPCR master mix (bimake.cn) and LightCycler 480 machine (Roche).

### SARS-CoV-2 viral RNA copy number assay

HEK293T-ACE2 cells were transfected with PVRL4 or vector plasmids using the pEI transfection reagent and infected with SARS-CoV-2 (MOI 0.25). The infected cells were harvested in Trizol at 24 h post-infection and RNA was isolated by standard isopropanol precipitation. RNA was reversed transcribed using iScript (BioRad) according to the manufacturer’s instructions with random hexamer as primers. The RT-qPCR analysis was done using the iCycler thermocycler (Bio-Rad). The primers used for RT-qPCR are SARS-CoV-2 NP (Forward 5’- TAATCAGACAAGGAACTGATTA − 3’, Reverse5’- CGAAGGTGTGACTTCCATG-3’). Expression values were normalized to ribosomal RNA L32 control and fold induction was normalized to untreated control. And the SARS-CoV-2 based experiment was performed at the UCLA BSL3 facility.

### Viruses binding, entry

Cells were infected with VSV (MOI = 5) or IAV (MOI = 5) and incubation at 4℃ for 1 h. For the binding assay, these cells were washed extensively with cold PBS twice and the cells were harvested to extract RNA to determine the viral copy number in the cell lysates through qRT-PCR assay. For the entry assay, after incubation at 4℃ for 1 h, the cells were washed with a low pH-PBS twice and then incubated with pre-warmed DMEM at 37℃ for another 30 min to internalize bound virion. After that, the cells were washed with acidic-PBS and the internalized viral particles were analyzed by qRT-PCR assay.

### Cell-to-cell fusion assay

For the VSV-G protein-mediated membrane fusion, HEK293T cells were co-transfected with pMD2G and PVRL4 or vector for 24 h. Moreover, HEK293T cells expressing PVRL4 or vector were mixed at 1:1 ratio and co-cultured with HEK293T cells expressing pMD2G for 6 h. Cells were then rinsed and incubated with acidic-PBS for 5 min. Subsequently, the cells were incubated in a 37℃ incubator for an additional 30 min. For the HSV-1 viral-mediated membrane fusion, cells were infected with HSV-1(MOI = 0.01) for 48 h. For SARS-CoV-2-spike-mediated cell-to-cells membrane fusion, HEK293T-ACE2 cells were co-transfected with SARS-CoV-2-spike-mCherry and vector or PVRL4 for 24 h. Moreover, HEK293T-ACE2 cells expressing PVRL4 or vector were co-cultured with HEK293T cells expressing SARS-CoV-2-spike-mCherry for 6 h at the ratio of 1:1. The formation of syncytia was observed using confocal imaging. The percentage of fusion was determined by normalizing the number of nuclei per syncytia under the experimental conditions to control.

### Endosomal acidification assay

Endosomal acidification was detected with a pH-sensitive dye (pHrodo Red dextran, Invitrogen, P10361) according to the manufacturer’s instructions with slight modification. First, WT and *PVRL4*^*−/−*^ A549 cells were added with 100 µg mL^− 1^ of pH-sensitive dye and Hoechst and then incubated at 4℃ for 15 min. Before taking images, cells were further incubated at 37℃ for 15 min and then cells were washed twice with PBS. Finally, PBS was added to cells and images were taken immediately with confocal microscope (Leica).

### Western blotting

Whole cell lysates were collected from cells extracted using lysis buffer containing 50 mM Tris-HCl (pH 7.5), 150 mM NaCl, 5 mM EDTA, 1% Triton X-100, 1 mM PMSF and 1×protease inhibitor cocktail (New Cell & Molecular Biotech Co.,Ltd). Proteins were separated on SDS–polyacrylamide gel electrophoresis (SDS-PAGE) and transferred onto polyvinylidene difluoride (PVDF) membranes. After blocking in 5% skimmed milk dissolved in the TBS containing 0.05% Tween 20, samples were immunoblotted with the indicated antibodies to measure the level of the expressed proteins.

### Immunofluorescence staining and confocal imaging

Cells were plated on Glass Bottom Dish (Cellvis) and were fixed with 4% paraformaldehyde for 15 min. And then, these cells were permeabilized with 0.2% Triton X-100/PBS for 10 min, before blocking with 5% skimmed milk for 1 h at room temperature. Then the nuclei were stained with Hoechst 33,342 at the appropriate dilutions. Cells were imaged on a confocal microscope (Leica).

### Pseudovirus production and infection

WT and variant SARS-CoV-2 pseudovirus with luciferase coding sequence were constructed previously in our lab [62]. To generate VSV pseudoviruses, HEK293T cells were transfected with pNL4-3-Luc HIV-1 NL4-3 △Env Vpr Luciferase and pMD2G, and the cells were further cultivated in growth medium for 48 h. The cells were infected with these pseudovirus for 24 h. Then relative luciferase activity was performed and normalized to the control cells to determine the pseudovirus quantity.

### Mice in vivo experiments

6–8 weeks old mice of WT and *Pvrl4*^−/−^ were injected intraperitoneally (i.p.) with VSV (1 × 10^8^PFU/g body weight). Liver, spleen and lung were separated for qRT-PCR to detect the VSV RNA expression after 36 h infection. Meanwhile, the lungs were stained with hematoxylin and eosin (H&E) for histopathologic analysis. For the WSN infection assay, 6–8 weeks old mice of WT and *Pvrl4*^*−/−*^ were infected intranasally (i.n.) with WSN (10,000 PFU). The animals were observed for 2 weeks, during which time weight loss and other signs of disease were assessed. The RNA level of NP in the lungs were measured by qRT-PCR at 2 and 4 days post-infection. And lungs were harvested at 2 days for the staining of H&E and immunohistochemistry staining the NP.

### Quantification and statistical analysis

All data were performed on GraphPad Prism 7. Statistical evaluation was performed by Student’s unpaired t test and two-way Student’s t test.Mouse survival curve analysis was calculated by the log rank test. Data are presented as Mean ± SEM, and *P* values are indicated by *P < 0.05, **P < 0.01 and ***P < 0.001.

### Electronic supplementary material

Below is the link to the electronic supplementary material.

Supplementary Material 1

## Data Availability

All study data are included in the article and SI Appendix.

## Abbreviations

PVRL4: Poliovirus receptor-like protein 4
IFN: Interferon
ISGs: IFN-stimulated genes
VSV: Vesicular stomatitis virus
HSV: Herpes simplex virus
IAV: Influenza A virus
SARS-CoV-2: Severe acute respiratory syndrome coronavirus
IFNAR1: IFNα/β receptor subunit1
