# Supplementary material for: Interferon-stimulated gene PVRL4 broadly suppresses viral entry by inhibiting viral-cellular membrane fusion
Source: Cell Biosci. 2024 Feb 17;14:23. doi: 10.1186/s13578-024-01202-y (PMC10873969; doi:10.1186/s13578-024-01202-y)
Supplement: Supplementary file 1 — Supplementary Material 1 [file 13578_2024_1202_MOESM1_ESM.docx]

**Supporting Information**


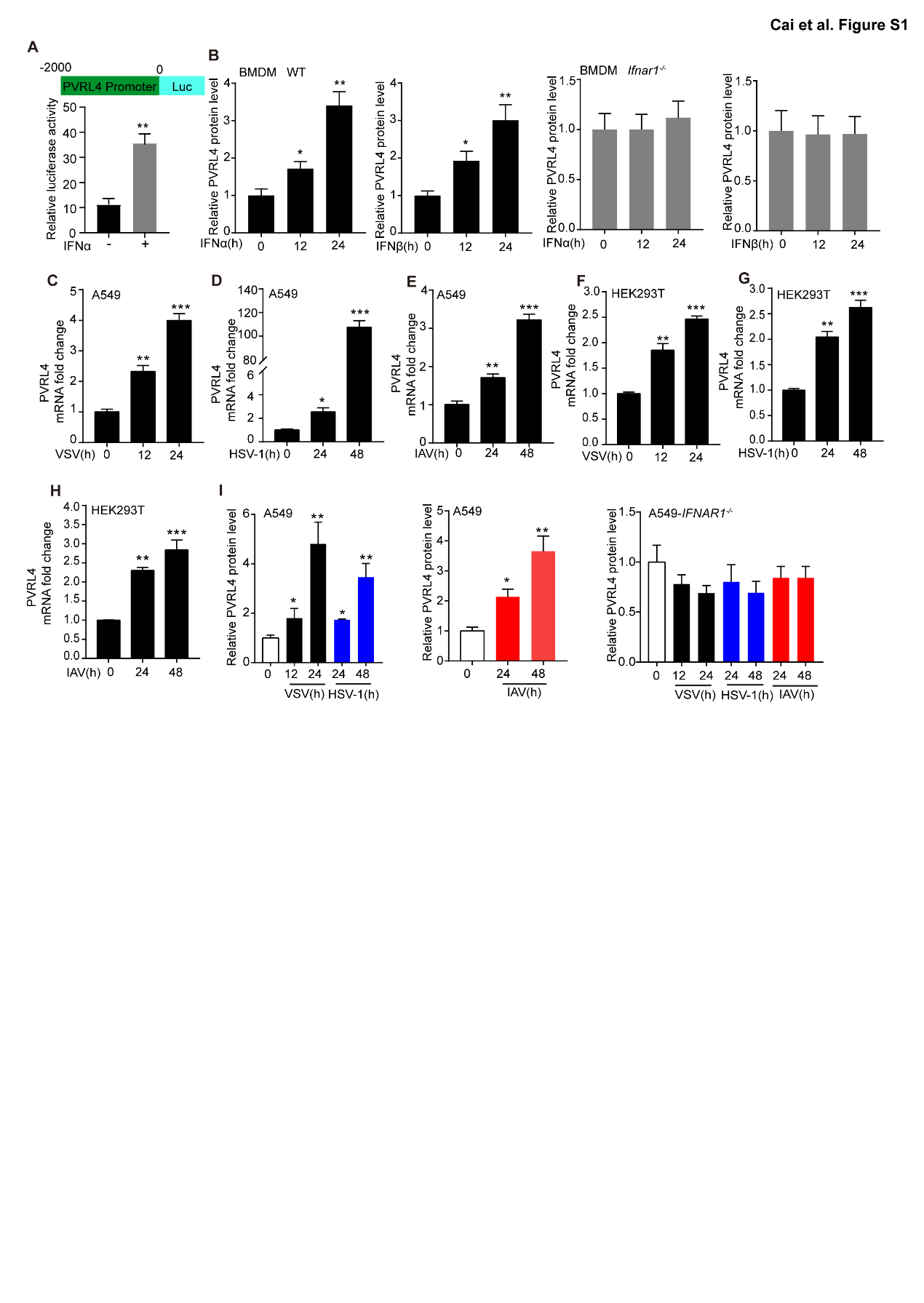


**Fig. S1. The expression of PVRL4 can be induced by various viruses.** (A) The model of construction of the pGL4.0-PVRL4 promoter. pGL4.0-PVRL4 promoter pGL4.0-PVRL4 promoter and Renilla luciferase plasmid were co-transfected into HEK293T cells. After 24h transfection, the cells were stimulated by IFN-α (1000U/mL) or not for 24h. Then, firefly luciferase and Renilla luciferase activities were detected using a dual luciferase reporter system. The ratio of firefly luciferase activity to Renilla luciferase activity was calculated. (B) Densitometry analysis of the data related in Fig. 1E. (C-H) qRT-PCR analysis of PVRL4 expression in A549 cells infected with VSV (C), HSV-1 (D) or IAV (E) for the indicated times as well as HEK293T cells (F-H). (I) Densitometry analysis of the data related in Fig. 1I. Mean ± SEM values from three independent experiments are shown. *p < 0.05, **p < 0.01, ***p < 0.001.


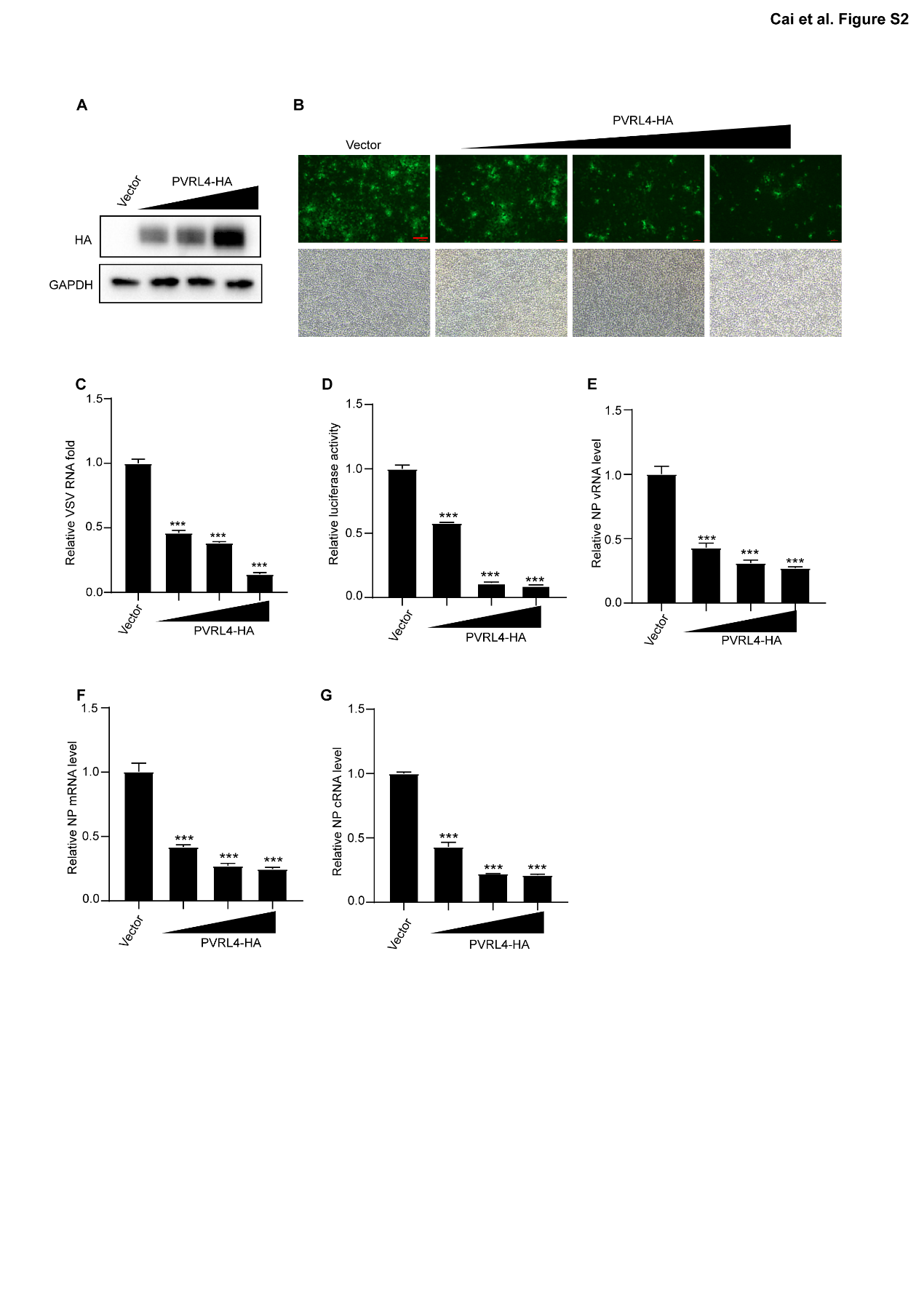


**Fig. S2. PVRL4 suppresses enveloped virus infection in a dose-dependent manner.** (A) Western blotting analysis of lysates from HEK293T cells transfected with increasing amounts of PVRL4-HA plasmids ((0, 100, 300, 500ng). (B and C) HEK293T cells were transfected with increasing amounts of PVRL4-HA plasmids for 24h. Then the cells were infected with VSV (MOI=0.01). The GFP were visualized by fluorescence microscopy (B) and the viral M RNA level was measured by qRT-PCR (C) after 12h infection. Scale bar, 100μm. (D) HEK293T cells were transfected with increasing amounts of PVRL4-HA plasmids for 24h. Then the cells were infected with HSV-1 (MOI=0.01), and the luciferase activity of cells was detected and normalized to the control after 24h infection. (E-G) HEK293T cells were transfected with increasing amounts of PVRL4-HA plasmids for 24h, then these cells were infected with IAV (MOI=0.01). After 24h infection, the viral NP vRNA, mRNA and cRNA level were measured by qRT-PCR. Mean ± SEM of three independent experiments. *p < 0.05, **p < 0.01, ***p < 0.001.


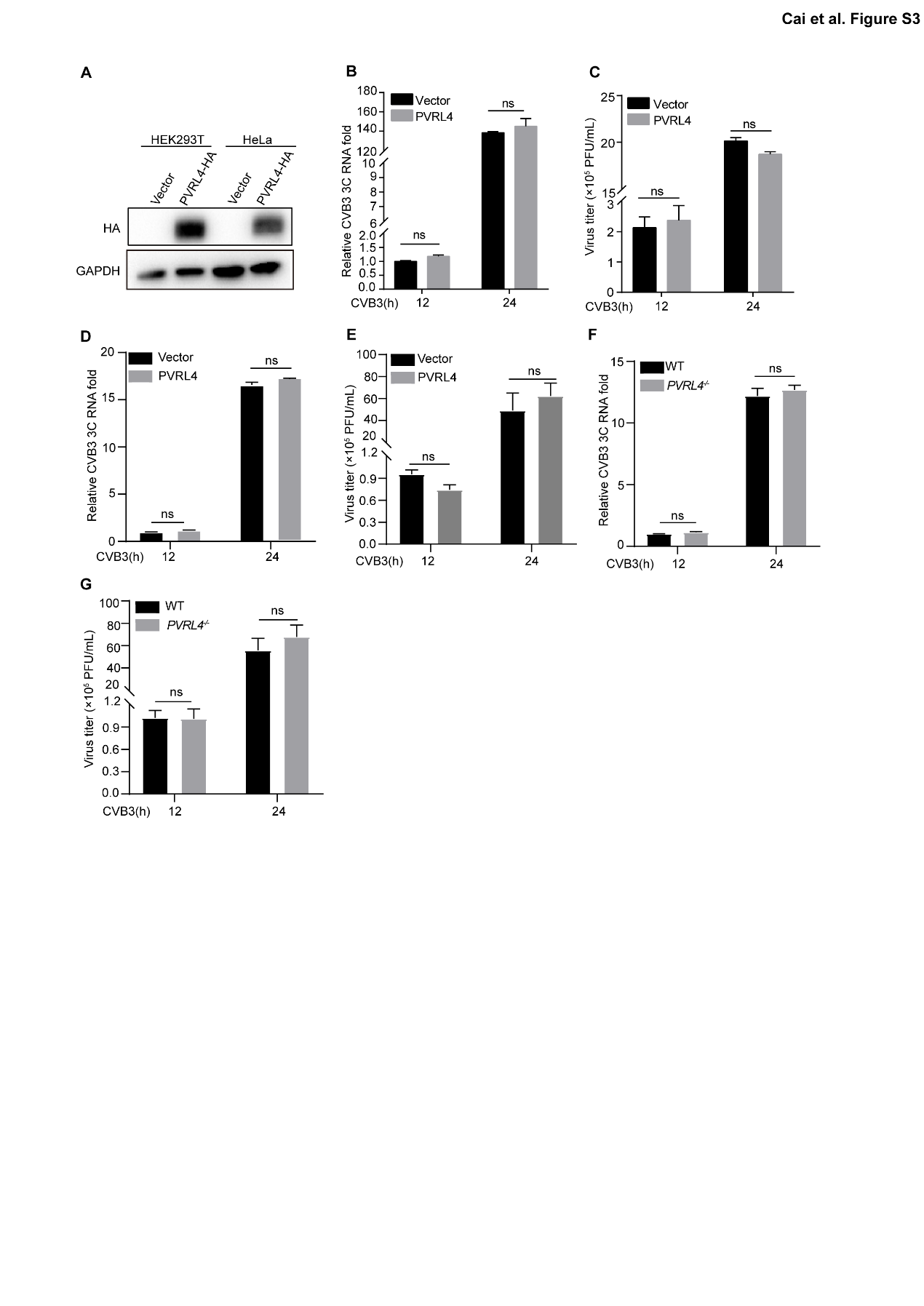


**Fig. S3. PVRL4 is failed to inhibit CVB3 infection.** (A)Western blotting analysis of lysates from HEK293T cells and HeLa cells transfected with PVRL4-HA or HA-expressing plasmids at 24h post-transfection. (B and C) HeLa cells were transfected with PVRL4-HA or contol vector for 24h and infected with CVB3 (MOI=0.01). The RNA level of CVB3 3C and the viral accumulation in the culture supernatants were measured by qRT-PCR and plaque assay, respectively, as the indicated times. (D and E) HEK293T cells were transfected with PVRL4-HA or contol vector for 24h and infected with CVB3 (MOI=0.01). The RNA level of CVB3 3C and the viral accumulation in the culture supernatants were measured by qRT-PCR and plaque assay, respectively, as the indicated times. (F and G) WT and *PVRL4^-/-^* HEK293T cells were infected with CVB3 (MOI=0.01). The RNA level of CVB3 3C and the viral accumulation in the culture supernatants were measured by qRT-PCR and plaque assay, respectively, as the indicated times. Mean ± SEM of three independent experiments. *p < 0.05, **p < 0.01, ***p < 0.001, two-tailed Student’s t test.


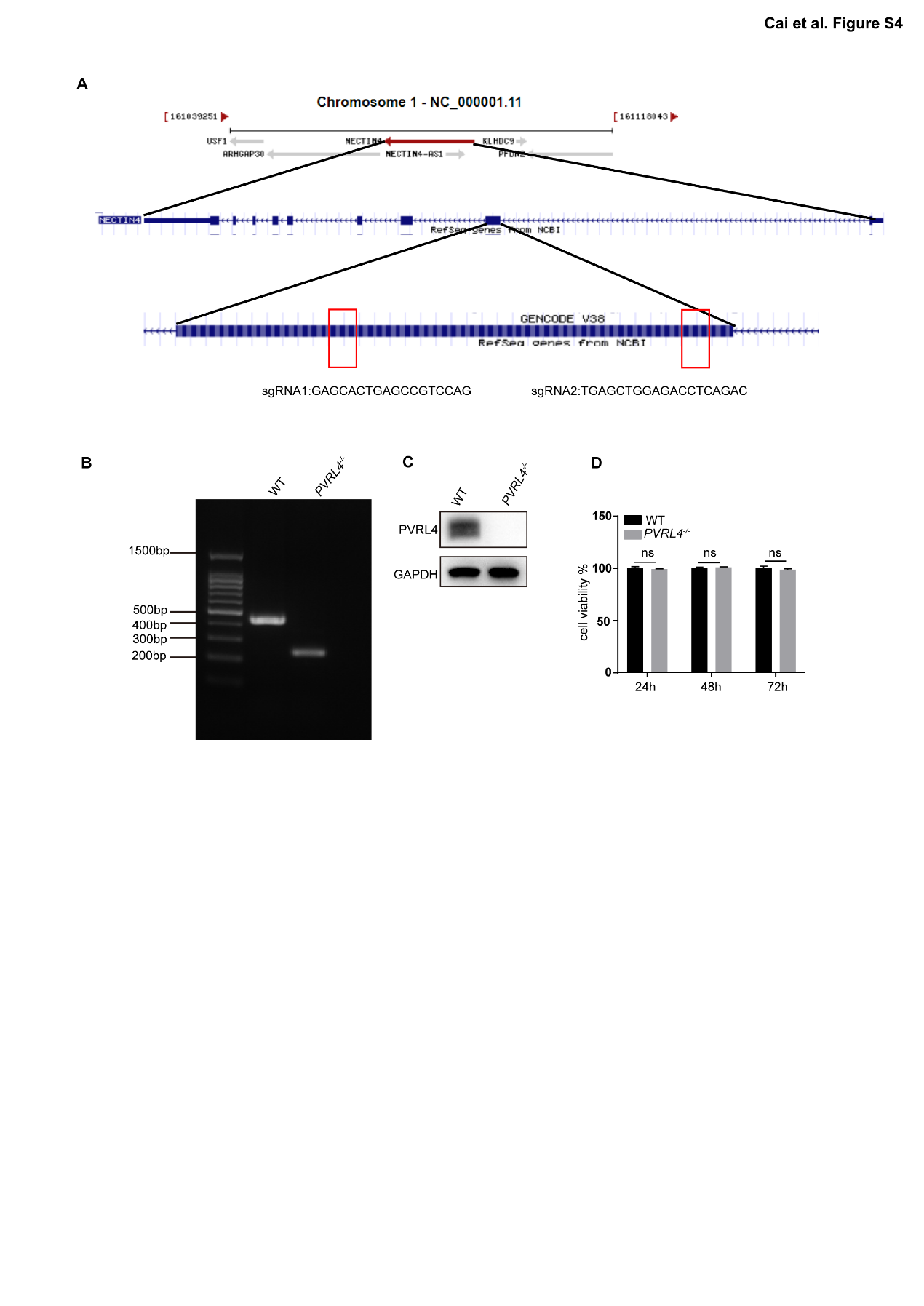


**Fig. S4. Construction of PVRL4 knockout HEK293T cell lines.** (A) Design of two sgRNAs targeting genome loci of Pvrl4 in HEK293T cells. (B) Deletion of ~200bp genomic DNA in *Pvrl4^-/-^* single clone was determined by PCR. (C) WT and *PVRL4^-/-^*HEK293T cell lines were immunoblotted for PVRL4. (D) Cell viability was assessed by CCK8 assay of WT and PVRL4^-/-^ HEK293T cells as the indicated times. Mean ± SEM of three independent experiments. *p < 0.05, **p < 0.01, ***p < 0.001, two-tailed Student’s t test.


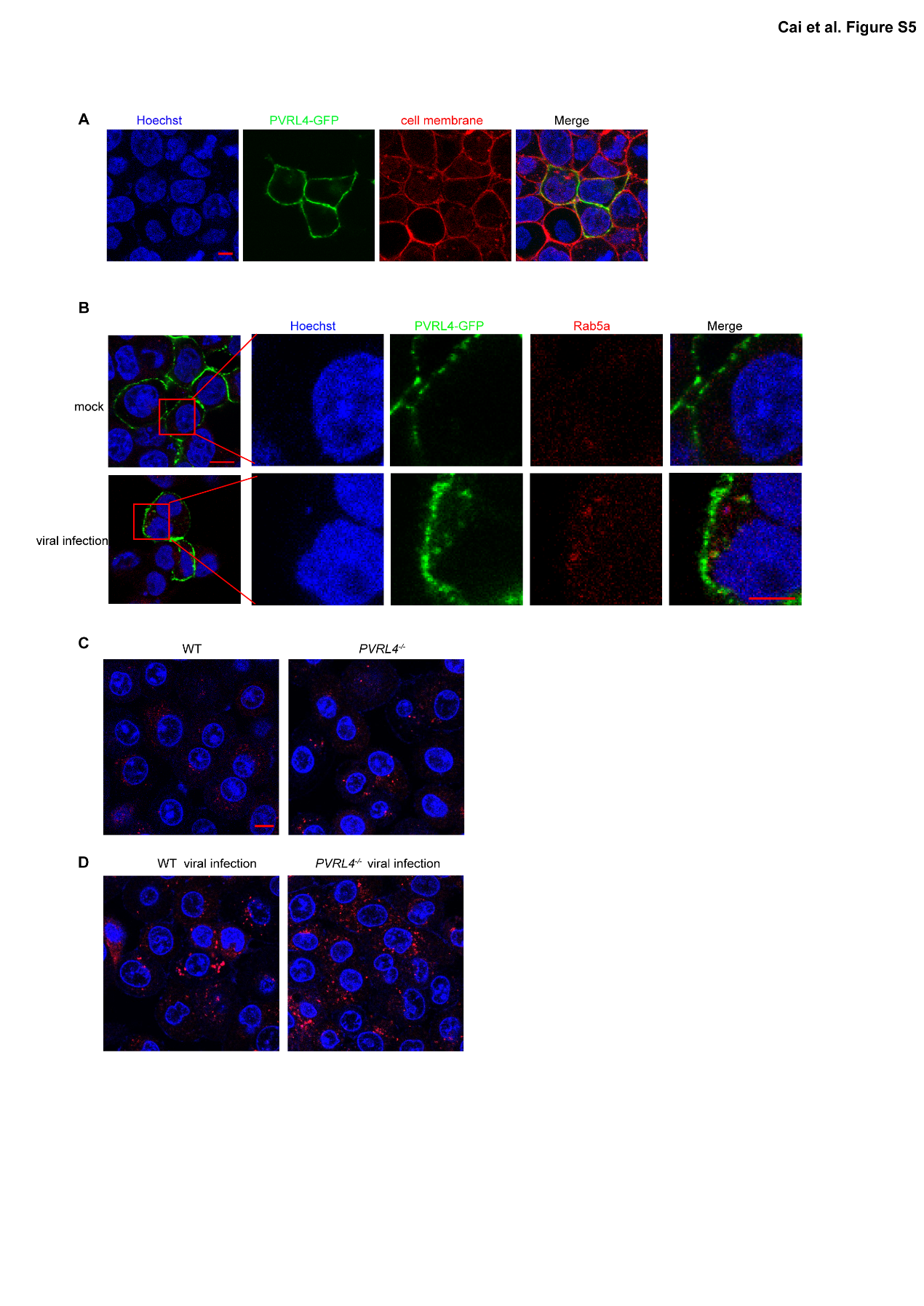


**Fig. S5. The cellular localization of PVRL4.** (A) HEK293T cells were transfected with PVRL4-GFP. After 24h transfection, cells were fixed and stained by cell membrane dye (red) and nuclear dye (blue). Scale bar, 10μm. (B) HEK293T cells were transfected with PVRL4-GFP for 24h. After that, the cells were infected with VSV for 8h or not, and then fixed and stained for early endosome (Rab5a) and nucleus (blue). Scale bar, 10μm, inset scale bar 5μm. (C and D) WT and *PVRL4^-/-^* A549 cells were treated by low pH indicator pHrodo^TM^ Red dextran (C). WT and *PVRL4^-/-^* A549 cells were infected with VSV for 8h and then treated by low pH indicator pHrodo^TM^ Red dextran (D). Red dots indicated the endosomes with low pH. Nuclei were stained with nuclear dye (blue). Live cell images were taken by confocal microscopeds. Scale bar, 10μm.


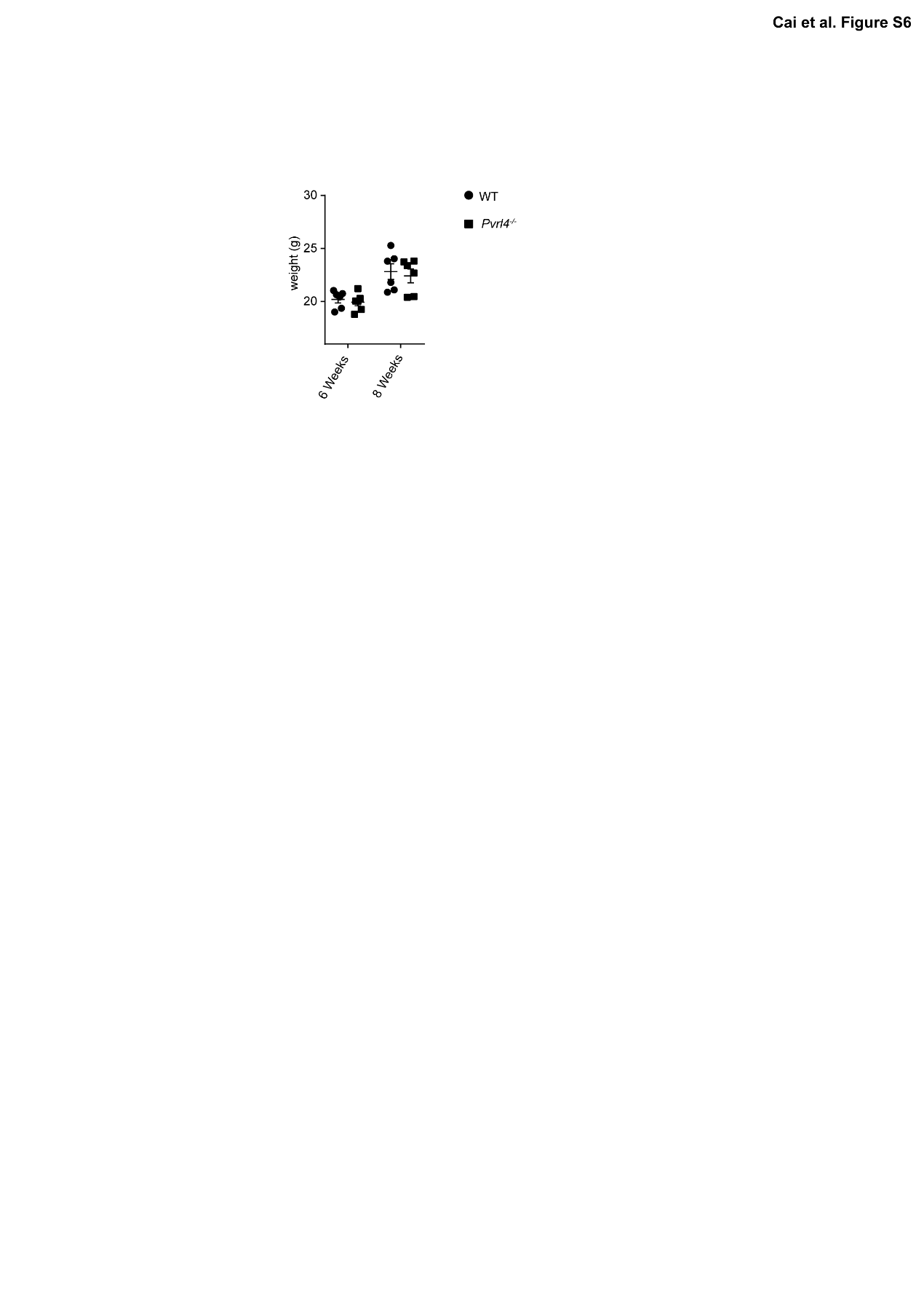


**Fig. S6. *Pvrl4^-/-^* mice were viable, normal in weight.** WT (n=6) and *Pvrl4^-/-^*mice (n=6) were weighed at 6 and 8 weeks.


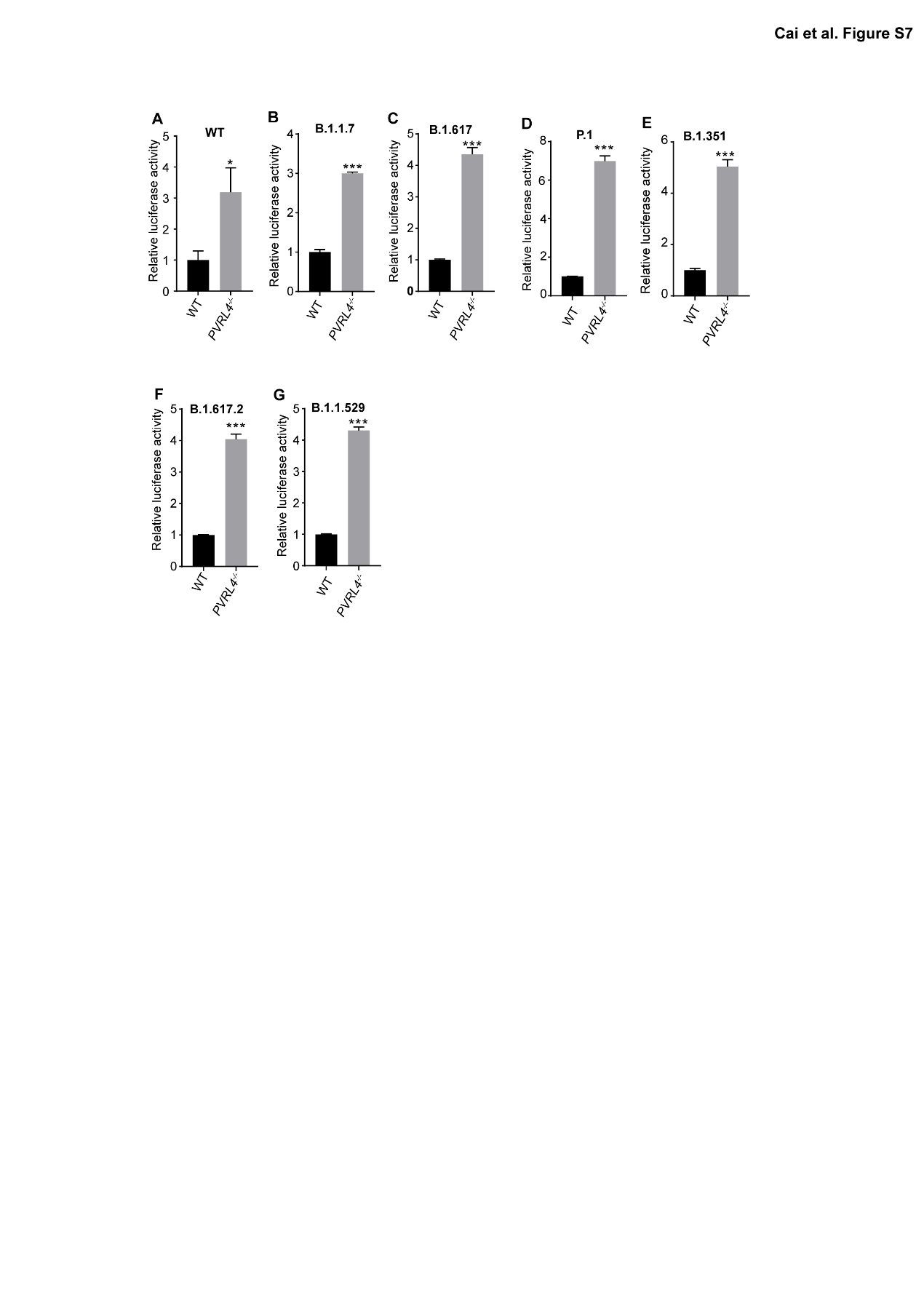


**Fig. S7. PVRL4-deficiency increases SARS-CoV-2 pseudovirus and various variants infection.** (A-G) WT or *PVRL4^-/-^* HEK293T cells were transfected with human ACE2 plasmid. After 24h transfection, the cells were infected with SARS-CoV-2 pseudovirus (A) or various variants (B-G) and the pseudovirus infection was quantified by luciferase activity at 24h post-infection. And the luciferase activities were normalized to the WT cells. Mean ± SEM of three independent experiments. *p < 0.05, **p < 0.01, ***p < 0.001, two-tailed Student’s t test.

**
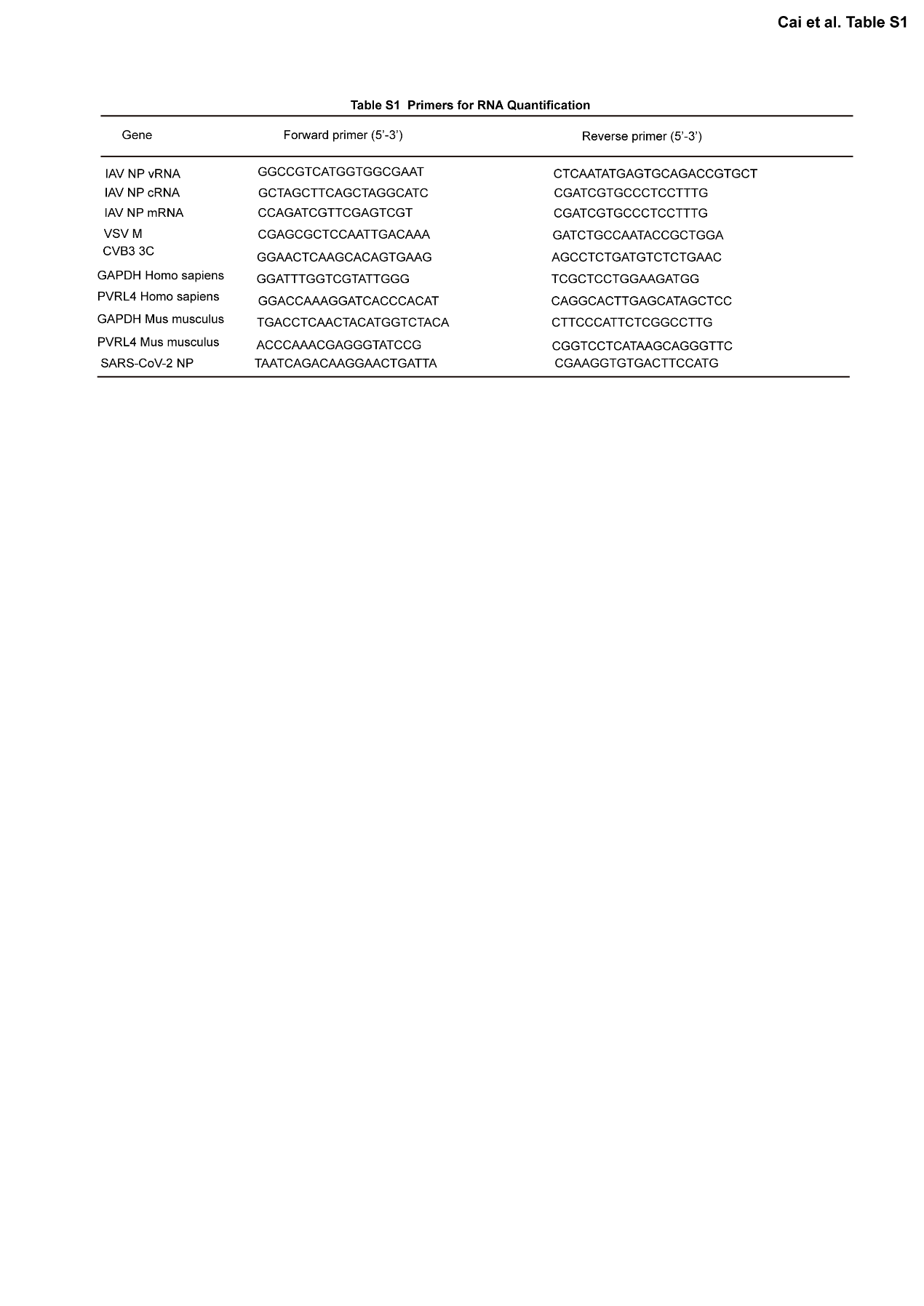
Table S1 Primers for RNA quantification.**
